# Comprehensive analysis of silicon impact on defense and metabolic responses in rice exposed to herbivory stress

**DOI:** 10.3389/fpls.2024.1399562

**Published:** 2024-05-30

**Authors:** Dandy Ahamefula Osibe, Yuko Hojo, Tomonori Shinya, Namiki Mitani-Ueno, Ivan Galis

**Affiliations:** ^1^ Institute of Plant Science and Resources, Okayama University, Kurashiki, Japan; ^2^ Department of Plant Science and Biotechnology, University of Nigeria, Nsukka, Nigeria

**Keywords:** rice (Oryza sativa), mineral, defense, metabolites, herbivore

## Abstract

Silicon (Si) uptake is generally beneficial for plants that need protection from insect herbivores. In pursue of mechanisms involved in Si-mediated defense, we comprehensively explored the impact of Si on several defensive and metabolic traits in rice exposed to simulated and real herbivory of *Mythimna loreyi* Duponchel larvae. Hydroponic experiments showed that Si-deprived rice supplemented with Si 72 h prior to insect infestation were similarly resistant to larvae as plants continuously grown in Si-containing media. Both Si and herbivory altered primary metabolism in rice, including the levels of several sugars, amino acids, and organic acids. While the accumulation of sugars was generally positively correlated with Si presence, multiple amino acids showed a negative correlation trend with Si supplementation. The levels of secondary metabolites, including isopentylamine, *p*-coumaroylputrescine and feruloylputrescine, were typically higher in the leaves of Si-supplemented plants exposed to herbivory stress compared to Si-deprived plants. In addition, simulated herbivory treatment in Si-supplemented plants induced more volatile emissions relative to Si-deprived plants, which was consistent with the increased transcripts of key genes involved in volatile biosynthesis. In ecological interactions, Si alone did not affect the oviposition choice of *M. loreyi* but gravid females showed a significant preference for simulated herbivory-treated/Si-deprived compared to Si-supplemented plants. Our data suggest that apart from mechanical defense, Si may affect rice metabolism in multiple ways that might enhance/modulate defense responses of rice under herbivory stress.

## Introduction

In their habitats, plants are often confronted with the attacks of insect herbivores, which requires proper balancing of growth and defense responses (McMillan, 2023). Consequently, plants have evolved several mechanical and biochemical defense strategies; some of which are constitutive, and others inducible to counter herbivores and preserve their own fitness (Schuman and Baldwin, 2016). Mechanical defense includes various inherent structural traits, such as waxy cuticles, trichomes, hairs, and spines which collectively deter insects from feeding on plants (Mitchell et al., 2016; Kaur and Kariyat, 2023; Balakrishnan et al., 2024). Some plants are known to hyperaccumulate minerals from the soil that acts as a physical barrier to insect feeding (Korth et al., 2006; Hanley et al., 2007; Debona et al., 2017). For instance, *Medicago truncatula* Gaertn., mutants with reduced levels of calcium oxalate were more susceptible to damage by beet armyworms (*Spodoptera exigua* Hübner) compared to the WT lines (Korth et al., 2006). Needle-like calcium oxalate crystals (raphides) and cysteine protease exerted synergistic defensive function against insect Eri silkmoth (*Samia ricini* Donovan) (Konno et al., 2014). Similarly, impregnation of non-glandular trichomes with silicon was demonstrated as one of the essential traits in defense of rice (*Oryza sativa* L.) cv. Nipponbare against chewing insect herbivores (Andama et al., 2020).

Besides increasing the physical strength to insects *via* deposition on trichomes and beneath the leaf cuticles, several studies have also suggested that Si accumulation could be affecting the insect damage by modulating the levels of inducible chemical defenses in herbivore-exposed plants (Reynolds et al., 2016; Alhousari and Greger, 2018). For example, Si pretreatment enhanced the activity of several plant defense-related enzymes after insect attack (Ye et al., 2013; Han et al., 2016). Such enzymes are often associated with the biosynthetic pathways for various types of defensive secondary metabolites, including various types of phenolics and momilactones (Fawe et al., 1998; Ahanger et al., 2020). Previously, we characterized several novel herbivore-induced metabolites in rice, which is a typical hyperaccumulator of Si (Alamgir et al., 2016; Aboshi et al., 2021); however, whether Si could be mediating the content of these metabolites in rice defense remains unknown.

In addition to specialized metabolites, Si application altered primary metabolism in unstressed rice by inducing the amino acid remobilization (Detmann et al., 2012). Under abiotic stress conditions, such as iron toxicity and drought, Si application enhanced photosynthesis (dos Santos et al., 2019), carbohydrates and amino acid accumulations (Hajiboland et al., 2017). The impact of Si on primary metabolism in plants under biotic stress was mainly explored in plant-pathogen systems, especially with the help of transcriptomics. For instance, Si treatment alleviated the down-regulation of genes involved in primary metabolism in Arabidopsis inoculated with powdery mildew fungus (Fautex et al., 2006). In addition, Si pretreatment increased the photorespiration rates in brown spot-infected rice plants, a mechanism thought to protect the photosynthetic machinery of plants under the stress conditions (Van Bockhaven et al., 2015). Although significant alterations in primary metabolism of plants also occur during herbivory stress (Zhou et al., 2015), possible effect(s) of Si on such metabolism in plants exposed to insect herbivores remain to be explored in detail (Frew et al., 2018).

After herbivore attack, in addition to direct defense metabolites, plants also produce volatile organic compounds (VOCs), such as terpenoids, green-leaf volatiles and aromatic compounds, which can either be directly toxic or indirectly protect plants by recruitment of natural enemies of herbivores, when retained or released from plants, respectively (Aljbory and Chen, 2018; Zhou and Jander, 2022). Previously, Si treatment was shown to affect the release of herbivory-induced volatiles (HIPVs) in herbivore-infested rice, and these alterations were ecologically important in attracting the natural enemies of herbivores to attacked plants (Liu et al., 2017). While the control of HIPV emissions in rice involves both biosynthesis and/or release mechanisms (Mujiono et al., 2021), which of these are modulated by Si application is not well understood. In addition, HIPVs are well known to serve as danger and/or host-location cues for ovipositing females of herbivorous insects (Zakir et al., 2013; Achhami et al., 2021). So far in maize, Si supplementation did not alter the oviposition choice of *S. exigua* (Leroy et al., 2022) but suppressed the *S. frugiperda* JE Smith oviposition on Si-enriched plants (Pereira et al., 2021).

In this study, we aimed for a more comprehensive approach to evaluate both defensive and metabolic roles of Si in rice plants under herbivory stress, represented by real herbivory of generalist chewing herbivore *Mythimna loreyi* (MYL), and simulated herbivory treatment (WOS), when mechanical wounds were treated with the oral secretions from MYL. Short-term Si uptake experiments in hydroponic system were used to monitor the distribution of Si in rice leaves, including local and systemic leaves after herbivore attack. The impact of Si on rice metabolism was analyzed by monitoring the accumulation of several primary and secondary metabolites, including HIPVs, in Si-supplemented and Si-deprived rice plants exposed to insect herbivory. By measuring the transcripts levels of genes putatively associated with HIPV biosynthesis in rice, we further attempted to elucidate the possible mechanisms involved in Si-modulated HIPV emissions in rice. In summary, our results further deepen the understanding of defensive and metabolic roles, and ecological implications, of Si in rice challenged with insect herbivores.

## Materials and methods

### Plant materials and growth conditions

Hydroponically-grown WT rice plants (*O. sativa* cv. Nipponbare) were used in all experiments, except in herbivore performance assay where Si-transporter-deficient Nipponbare rice mutants, *lsi1* and the corresponding WT plants were grown in pots with paddy field soil. Nipponbare was selected for this study because it is a reference rice cultivar with well characterized Si accumulation mechanisms (Ma et al., 2007a). Seeds of *lsi1* mutant were obtained from Dr. Jian Feng Ma, Okayama University, Japan. All seeds were soaked in water and kept in the dark for 2 d at 30°C. In hydroponic growing system, seeds were germinated for 4 d on a net floating in a solution containing 0.5 mM CaCl_2_ as described previously (Ma et al., 2002). The seedlings on net were then transferred to a half-strength Kimura B solution containing 0.5 mM Si as silicic acid. Silicic acid was prepared using potassium silicate passed through a cation-exchange resin (Amberlite IR-120B, H + form, Organo, Tokyo). For Si-depleted treatments, silicic acid was not added to Kimura B solution. About 2 weeks later, seedlings were transferred to 3.5 L pots, 50 seedlings each, supported by soft sponge inserted in pot cover with 2 cm round holes. Nutrient solution was replaced once every 2 d. Four weeks after germination, seedlings were individually transferred to a 300-mL black plastic bottle containing Kimura B solution with or without Si. The solution was changed twice every week. Plants were grown in a cultivation room with a 14 h photoperiod and temperature of 28 ± 3°C. For soil culture, seedlings of WT (Nipponbare) and corresponding *lsi1* mutant were grown in pots with paddy field soil as described in Mujiono et al. (2020). Rice plants were used for most experiments 7-9 weeks after germination.

### Plant treatments

#### Real herbivory/MYL treatment

Loreyi armyworms, *M. loreyi* (Lepidoptera: Noctuidae) (abbreviated as MYL in following text) originally collected from rice in the paddy field (Kurashiki, Okayama Prefecture, Japan) were maintained in the laboratory on artificial pinto bean-based diet and rice leaf. Insects were reared at 25 ± 1°C under 16 L: 8D photoperiod. Neonates hatched from eggs on the rice leaves were transferred to artificial pinto bean-based diet (Gulzar and Wright, 2015) and maintained until pupation. Thereafter, pupae were kept in paper tissue for hatching in flight cages supplied with 10% diluted honey solution cups and rice seedlings serving as moth diet during mating period and oviposition substrate, respectively. Prior to use for bioassays, larvae at the 2^nd^-3^rd^ instar stage were pre-fed with rice seedlings overnight and subsequently starved for at least 3 h before placement on mature leaves of rice plants (defined as second fully expanded leaf after the youngest fully expanded leaf).

#### Mechanical wounding and oral secretions treatment

Mechanical wounds were made using a fabric pattern wheel on both halves of the treated leaf along the midvein, followed by treatment with 20 µL of water-diluted (3:1) oral secretions from MYL (WOS) evenly spread on the leaf surface by gentle rubbing. Oral secretions used in all experiments were prepared essentially as described in Shinya et al. (2016).

### Herbivore performance assay

Youngest fully expanded leaves of WT plants with or without Si amendments and *lsi1* mutant were individually infested with one pre-weighed 3^rd^ instar larva in clip cages (7.5 cm x 7.5 cm). All larvae were weighed at 2 d intervals until 6 d and the percentage mass gain on each plant was calculated. Thirty plants on each Si amendment regime were used for the assays except for the short-term Si supply performance assay where fifteen plants were used for each treatment.

### Analysis of Si uptake and accumulation

To observe the tissue-specific accumulation of Si upon exposure to herbivory, mature leaves of 7-weeks-old plants grown with or without Si were infested with 3^rd^ instar larvae in clip cages. After 48 h, the local leaves (second mature leaves) and the systemic leaves (youngest fully expanded and third old mature leaves) were harvested, frozen in liquid nitrogen and stored at -80°C until use for determination of Si concentration.

To examine the dynamics of Si uptake in rice seedlings exposed to either Si alone or in combination with insect infestation, we performed a short-term Si uptake experiment. Plants grown without Si for 7 weeks were either supplied with Si alone or in combination with herbivore treatments. Local and systemic leaves were sampled at designated time intervals (0, 3, 6, and 24 h) for Si analysis.

Determination of Si content in rice leaves was performed as described by Wei-min et al. (2005) with some modifications. Frozen leaf samples were pulverized into fine powder over liquid nitrogen and 100-150 mg aliquots were transferred into a 5-mL Eppendorf tubes, and oven dried at 60°C for 48 h. After oven drying, each tube was added 750 µL of 50% NaOH and gently vortexed. The tubes were then covered with screw caps and autoclaved at 121°C and 138 kPa for 1 h. Ten µL aliquots from the digested sample were diluted with 157 µL of water, thereafter 20 µL of the diluted solution was transferred to 1.5 mL tube and combined stepwise with 600 µL of 20% (vol/vol) acetic acid and 200 µL ammonium molybdate (54 g/L, pH 7.0). Samples were vortexed and kept at room temperature for 5 min before the addition of 100 µL of 20% (wt/vol) tartaric acid and 20 µL of freshly prepared reducing agent (40 mg Na_2_SO_3_, 8 mg 1-amino-2-naphtol-4-sulfonic acid, 500 mg NaHSO_3_ in 5 mL of milli-Q water). Final reaction volume was adjusted to 1 mL with acetic acid before incubating at room temperature for 30 min. Absorbance was measured at 650 nm after 30 min incubation at room temperature using a spectrophotometer.

### Visualization of Si deposition in rice leaves by SEM-EDX

Scanning electron microscopy (SEM) coupled with energy dispersive X-ray spectrometry (EDX) was used to observe the pattern of deposition of Si in rice leaves exposed to either Si alone or in combination with insect feeding. Rice seedlings (7-weeks-old) grown in the absence of Si were transferred to a Kimura B solution containing 0.5 mM Si alone or in combination with herbivore exposure for 24 h. Leaf discs (4 mm) collected from mature leaf of herbivore-fed and control plants were placed on a double-sided adhesive tapes and oven-dried at 40°C for at least 24 h before SEM imaging. The abaxial surface of the leaf discs was used for SEM observation on Miniscope TM 3000 (Hitachi High-Technologies, Tokyo, Japan) at 15 kV. Scanning electron micrographs obtained at 500-fold magnification were used to map Si deposition pattern on leaf surface using Swift ED3000 X-ray microanalyzer coupled to the microscope.

### Leaf photosynthetic parameters

Leaf gas exchange rates (G_s_ and T_r_) and photosynthetic activity (P_n_) were measured using a portable gas exchange analysis system LI-6400XT coupled with a leaf chamber Fluorometer 6400-40 (LI-COR Biosciences, Lincoln, NE, USA). The ratio of *P_n_* and *G_s_* was calculated as the intrinsic water use efficiency (iWUE). The temperature of the leaf chamber was set at 28°C to match with the temperature in the plant growth room at the start of measurement. During measurement, light intensity and CO_2_ concentration in the leaf chamber were maintained at 1000 µmol m^-2^ s^-1^ and 400 ppm, respectively. The leaf was allowed to acclimate for 30 min after enabling the light source of LI-6400XT, before each measurement. Mature leaves (second fully expanded leaf) were used for the measurements. WOS treatments were performed as described above on mature leaves. After 24 h, leaf photosynthetic activity was measured on the WOS-treated leaf.

### Metabolites and phytohormone analyses

Sampled leaf tissues (70-150 mg fresh mass) were ground to fine powder in liquid nitrogen before adding 4-5 ceramic beads for sample homogenization by reciprocal shaking using a FastPrep 24 instrument (MP Biomedicals, Santa Ana, CA, USA). Sample primary metabolites extraction, quantification, and analysis with an Agilent 7890A-GC/240-MS instrument (Agilent Technologies, Santa Clara, CA, USA) were performed as described in Raviv et al. (2020). For the identification of primary metabolites after GC-MS analysis, commercially available authentic standards for each compound were used for comparison of retention times and fragmentation patterns, together with the information from the NIST Mass Spectral Library version 2.0g build 2011 (National Institute of Standards and Technology, Gaithersburg, MD, USA). Secondary metabolite content in leaf samples was measured with 6410 Triple Quadrupole LC/MS (Agilent Technologies) after extraction and purification as described previously with slight modifications (Tanabe et al., 2016). For secondary metabolite sample purification with C18 column, supernatants from sample extractions were combined and diluted with 84 mM ammonium acetate buffer (pH 4.8) to a final methanol concentration of 20% (v/v) before purification with a C18 solid-phase extraction column (Bond Elut C18, Agilent Technologies). The amount of secondary metabolites in the samples was determined after LC-MS analysis using synthetic standards of phenolamides prepared in Alamgir et al. (2016) and authentic isopentylamine (Tokyo Chemical Industry Co., Ltd., Japan). Phytohormone analysis was essentially carried out as described in a previously published method (Fukumoto et al., 2013). Endogenous phytohormone amounts were quantified using deuterium-labeled internal standards from commercial sources (d6-ABA, Funakoshi Co. Ltd. Japan; d4-SA, Toronto Research Chemicals Inc., Canada) or d3-JA, d3-JA-Ile donated by Dr. H. Matsuura (Hokkaido University).

### Quantification of headspace volatile organic compounds

A headspace VOC collection system previously described in Sobhy et al. (2017) was used to collect and quantify the VOCs released from rice plants grown with or without Si amendment and treated with or without WOS. For the WOS-treated sets, the youngest fully expanded leaf and the mature leaf (second fully expanded leaf) were subjected to WOS treatment as described earlier, while the control set remained unwounded. Briefly, for VOC collection, treated or control plants grown hydroponically in 300-mL black plastic bottles were individually inserted in an acrylic cylinder (50 cm high x 15 cm internal diameter) with an inlet port that allowed purified air into the cylinder, and an outlet port at the top fitted with volatile traps containing two MonoTrap devices (cylindrical monolithic adsorbents; GL Sciences Inc., Tokyo, Japan). After 24 h of trapping, Mono Traps were transferred to GC vials and eluted by sonication with 300 µL dichloromethane (DCM; FUJIFILM Wako Pure Chemical Corporation, Osaka, Japan) spiked with 400 ng tetralin (1,2,3,4-tetrahydronaphthalene; FUJIFILM Wako Pure Chemical Corporation) as an internal standard. Eluted samples were analyzed on an Agilent 7890A-GC/240-MS instrument (Agilent Technologies) using previously optimized instrument parameters (Sobhy et al., 2017). For the identification of VOCs after GC-MS analysis, commercially available authentic standards for each compound were used for comparison of retention times and fragmentation patterns, together with the information from the NIST Mass Spectral Library version 2.0g build 2011 (National Institute of Standards and Technology, Gaithersburg, MD, USA).

### Quantitative RT-PCR

Gene expressions were quantified with quantitative RT-PCR as previously described in Fukumoto et al. (2013). Gene-specific oligonucleotide primers used for qRT-PCR are outlined in the **Supplementary Table 1**.

### Oviposition preference bioassays

All oviposition preference assays were performed under outdoor conditions in a wire-mesh screen house. The experimental setup consisted of rice plants randomly arranged on tables in the screen house such that each plant with Si amendment was placed at distance of 50 cm from those without Si amendment. For each test, 20 adult females and 5 adult males of MYL moths were released in screen house containing 24 plants (12 plants each for each treatment either Si-treated or non-treated plant or Si regimes with WOS). Diluted honey solution was provided *ad libitum* as food source. To determine oviposition preference, the number of plants with egg masses for each treatment was counted after 1 and 2 d, and an oviposition preference percent: {(no. of plants with egg masses/total no. of plants) x 100} was calculated. The bioassay was independently performed with rice plants grown hydroponically with or without Si, and then plants grown with or without Si treated with WOS to simulate herbivory stress. For the simulated herbivory stress sets, the youngest and mature fully expanded leaves were subjected to WOS treatment in the screen house after which the MYL moths were released and oviposition preference was determined after 1 and 2 d. Each set was repeated at least 5 times with new sets of plants and insects used for each repeat.

### Statistical analyses

Data were first examined for normality using the online version of Shapiro-Wilk test (www.statskingdom.com/shapiro-wilk-test-calculator.html), and when not normally distributed, logarithmic transformations of data were used before statistical analysis. Statistical differences between pairs of datasets were determined using Student’s t-test (Microsoft Excel), and multiple samples were compared by analysis of variance (ANOVA) in *multcomp* package in R version 4.3.2 (R Core Team, 2023). The impact of Si and herbivory on the identified primary metabolites was visualized with Pearson’s correlation, and principal component analysis (PCA) packages in MetaboAnalyst 6.0 (http://www.metaboanalyst.ca).

## Results

### Supply of silicon enhances rice resistance to MYL

**Figure 1:**
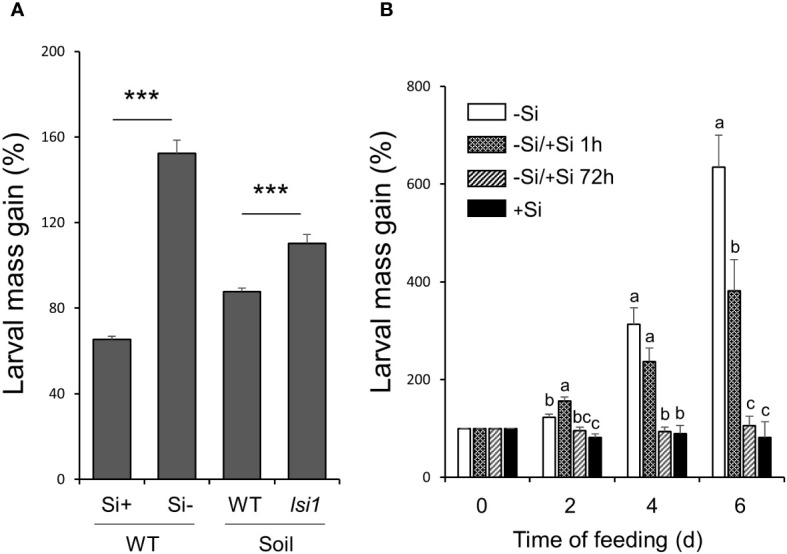
Gain in mass of *M. loreyi* fed on rice plants. **(A)** Nipponbare (WT) rice plants grown in hydroponic solution supplied with or without 0.5 mM Si and Si transporter deficient Nipponbare rice mutant (*lsi1*) grown in pots with paddy soil; **(B)** WT rice plants grown in hydroponic solution under different Si regimes:(1) grown without Si (-Si), (2) supplemented with Si 1 h before setting larvae on plants (-Si/+Si 1h), (3) supplemented with Si 72 h prior to herbivory (-Si/+Si 72h) or (4) continuously supplied with Si (+Si). Youngest fully expanded leaves of all plants were individually infested with third instar larvae. All larvae were weighed after 2 d for **(A)** or at intervals of 2 d for **(B)** and the percentage mass gain was calculated. Data are mean ± SE (n=30 for 1A, n=15 for 1B). Statistical differences between Si treatments were analyzed by Student’s t-test (***P<0.001). Different letters **(a-c)** indicate significant differences (P ≤ 0.05) between different Si regimes at each time point according to ANOVA followed by Tukey’s HSD test.

To assess the overall importance of Si in rice resistance to chewing herbivores, we first performed a bioassay using larvae of MYL that fed on hydroponically-grown Nipponbare (WT) rice plants. The plants in Kimura B culture media were supplied with or without 0.5 mM silicic acid, and replenished with a fresh media in twice a week time interval. MYL larvae fed on 7-week-old WT plants without Si increased their mass to 152.2% at 2 d of infestation, whereas larvae fed on Si-supplemented WT plants retained on average only mass of 65.3% relative to initial conditions (**Figure 1A**). To further confirm that the observed herbivore growth patterns reflect the accumulation of Si in the rice leaves, we compared MYL performance on Si-transporter-deficient Nipponbare *lsi1* mutants, and their corresponding WT plants grown in pots with paddy field soil. Again, MYL larvae grew better on the *lsi1* mutant plants, which was significant at 2 d of insect infestation when compared to similarly grown WTs (**Figure 1A**). These results corroborated a previously known defensive role of Si in rice against chewing herbivores that requires impregnation of rice tissues with silicon. We then examined the time interval required for Si to establish resistance against MYL larvae in rice leaves. WT plants were kept in Si-free hydroponic solution for 7 weeks, and then treated with different Si regimes before starting herbivore performance assays (**Figure 1B**): (1) grown without Si (-Si) (2) supplemented with Si 1 h before setting larvae on plants (-Si/+Si 1h) or (3) supplemented with Si 72 h prior to herbivory (-Si/+Si 72h). As before, plants continuously supplied with Si (+Si), used as a positive control, strongly inhibited MYL performance; interestingly, when the plants were supplied with Si 72 h prior to MYL exposure, we observed a similar level of resistance to that of the positive control rice plants (+Si) (**Figure 1B**). However, plants treated with Si 1 h prior to larvae exposure did not show any significant reduction in the larval growth until after 4 d of infestation. These results indicated a 3-4 d period as a minimum time necessary for Si to establish its defensive role in rice, at least under the model conditions when the plants were first deprived of Si in the culture media.

### Si accumulation and deposition patterns in rice leaves exposed to herbivory stress

**Figure 2:**
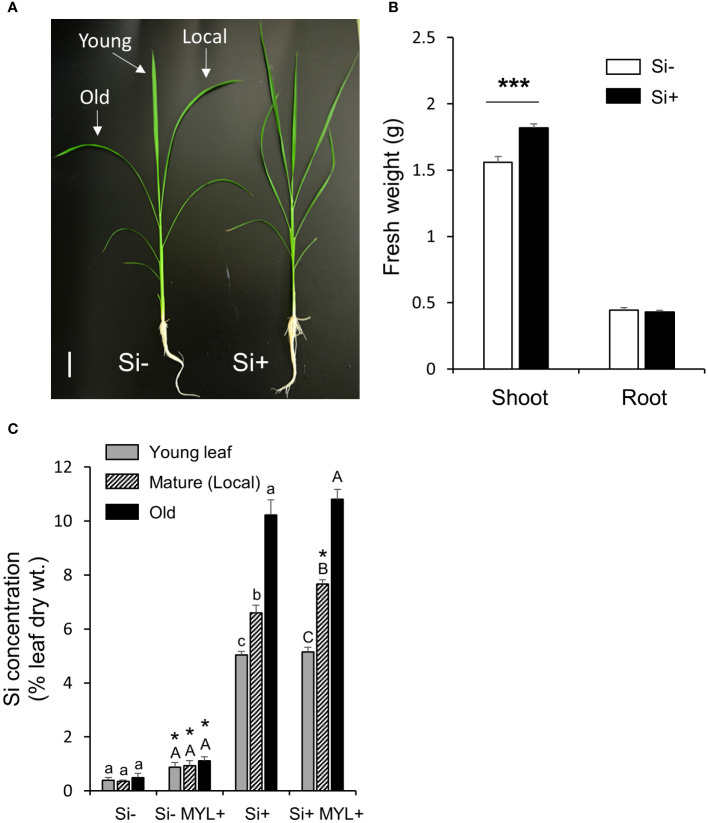
Silicon accumulation and impact on growth of rice plants. **(A)** Growth phenotype; **(B)** Shoot and root fresh weight (n=15); **(C)** Accumulation of Si in rice leaves after 48 h exposure to *M. loreyi* feeding (MYL). WT rice plants were grown in hydroponic solution supplemented with or without 0.5 mM Si for 7 weeks. Statistical differences between treatments (with or without herbivory) were analyzed by Student’s t-test (*P<0.05; ***P<0.001). Different letters (a-c or A-C) indicate significant differences (P ≤ 0.05) between different leaf ages determined separately for each group (Si or herbivory treatments) according to ANOVA followed by Tukey’s HSD test. Scale bar indicates 5 cm.

**Figure 3:**
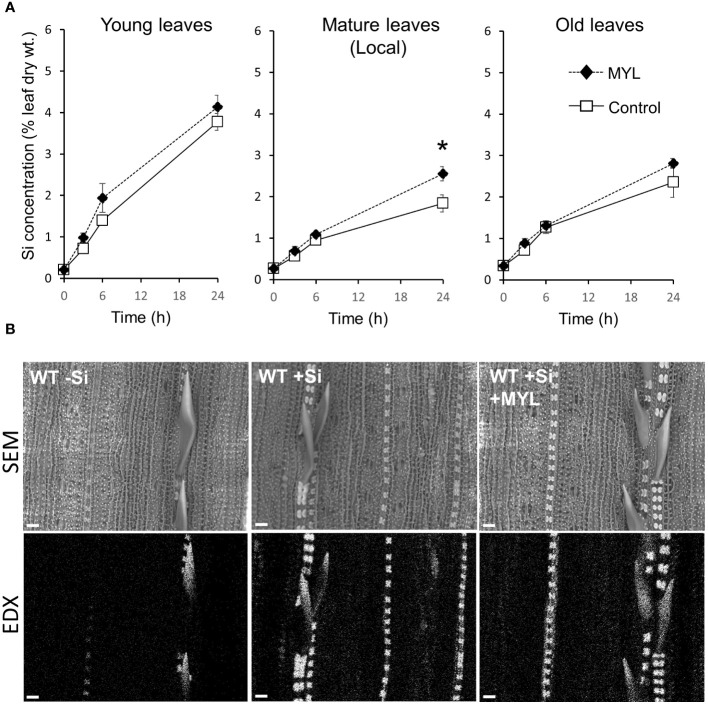
Distribution and deposition patterns of Si in rice exposed to herbivory stress. **(A)** Si concentration in different rice leaves after short-term exposure to Si and *M. loreyi* feeding (MYL; n=5); **(B)** Scanning electron micrographs with the corresponding Si EDX map images of mature leaf surface of rice plants. WT rice plants (7 weeks) grown in Si-free hydroponic solution were transferred to a hydroponic solution containing 0.5 mM Si and exposed to *M*. *loreyi* feeding for 24 h. At indicated time intervals, mature herbivore fed (local), and systemic (young, old) untreated leaves were collected for Si determination. After 24 h exposure to either Si alone or in combination with herbivore feeding, 4 mm leaf discs were excised from mature leaf of herbivore fed and control leaves and subjected to SEM-EDX imaging. Scale bars indicate 20 µm. Statistical differences between treatments (Control and herbivory) were analyzed by Student’s t-test (*P<0.05; no symbol, not significant).

Taking into account a gradual build-up of anti-herbivore Si-dependent defense, herbivores may be affecting the distribution of Si in the insect exposed plants, although most of the mineral is likely to remain immobile due to fixation in specialized rice cells, such as silica bodies and impregnated trichomes (spikes). We first analyzed the leaf Si distribution using a set of hydroponically grown rice plants in the continuous presence and absence of Si, which were exposed to feeding of MYL larvae for 48 h. Notably, regardless of herbivory, hydroponically grown plants without silica showed a reduced fresh mass of the aboveground parts (**Figures 2A, B**). As expected, plants in Si-containing culture media accumulated large amounts of Si (5-10% dry weight) in all tested leaves, compared to the non-Si treated plants (**Figure 2C**). Notably, Si accumulation differed with leaf age. Exposure to insect feeding for 48 h resulted in the significantly higher accumulation of Si, but only in the locally-fed leaves (**Figure 2C**). A short-term Si uptake experiment, wherein the non-Si treated plants were either exposed to Si alone or in combination with insect infestation, showed somewhat different pattern. Firstly, higher Si distribution to young, yet expanding leaves was observed, compared to second (mature) and third (older mature) leaves (**Figure 3A**), suggesting a preferential flow of Si into newly developing tissues. Furthermore, Si accumulation tended to be higher in all leaves of MYL-exposed plants, whilst the difference was only significant in the locally exposed, i.e. second (mature) leaf 24 h after exposure to herbivory (**Figure 3A**). In addition, spatial distribution of Si in rice leaves after short term supply of Si was explored by SEM coupled to energy dispersive X-ray microanalysis (EDX) (**Figure 3B**). Si was similarly distributed in the control rice leaves and MYL-fed leaves after 24 h of Si uptake, namely, Si was deposited in silica bodies and hardened spikes. Notably, EDX was not able to capture the subtle quantitative differences observed in chemically-analyzed rice leaves with and without exposure to herbivory (**Figures 2C**, **3A**).

### Transcriptional responses of Si transporter genes to herbivory stress

**Figure 4:**
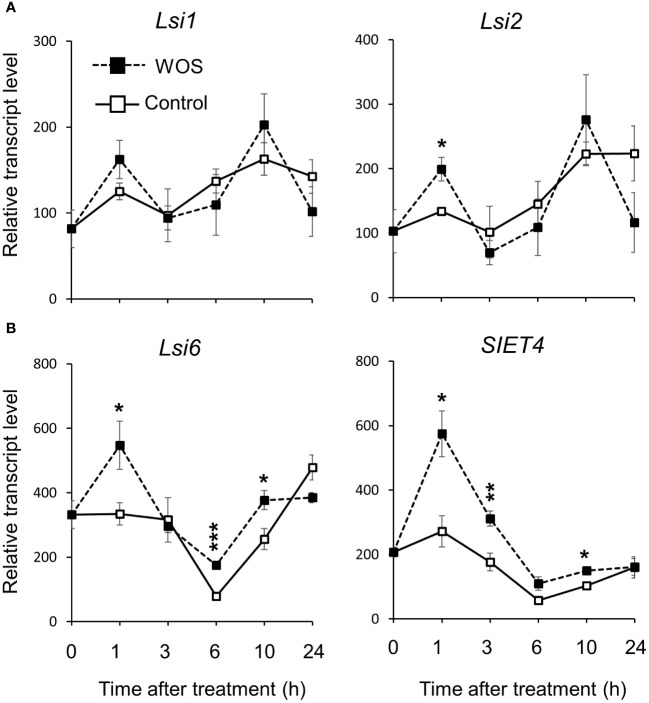
Relative transcript levels of Si transporter genes in WT rice plants after exposure to herbivory stress. Transcript levels of *Lsi1* and *Lsi2* in roots **(A)**, and of *Lsi6* and *SIET4* in fully expanded rice leaves (young and mature leaves; **(B)**. Gene expression was determined at indicated time intervals by qRT-PCR in 7-week-old WT rice plants before and after elicitation with wounds treated with oral secretions from *M. loreyi* (WOS). Data are mean ± SE (n=4). Statistical differences between treatments Control and WOS were analyzed by Student’s t-test (*P<0.05; **P<0.01; ***P<0.001 no symbol, not significant).

While *Lsi1* and *Lsi2* are required for Si uptake by roots (Ma et al., 2006; Ma et al., 2007b), *Lsi6* and *SIET4* have important roles in the distribution and deposition of Si in rice leaves (Yamaji et al., 2008; Mitani-Ueno et al., 2023). Because real herbivory experiments are difficult when precise timing is required, such as determination of early gene expression patterns, we used a simulated herbivory approach. In this method, mechanical wounds introduced by a fabric pattern wheel are treated with oral secretions (WOS) isolated from MYL larvae, after which the transcript levels of *Lsi1* and *Lsi2* in the roots, and *Lsi6* and *SIET4* in the leaves of Si-supplemented rice, untreated or exposed to herbivory, were analyzed. The *Lsi1* and *Lsi2* were only transiently induced in the roots 1 h after WOS elicitation (**Figure 4A**). In contrast, *Lsi6* and *SIET4* transcripts were clearly up-regulated 1 h after WOS treatment and gradually decreased, albeit still showing higher transcript levels relative to control untreated plants at time points between 3 and 10 h post treatment (**Figure 4B**, **Supplementary Figure 1**). These data show that distribution rather than additional uptake of Si may be regulated by herbivory stress to mount the effective plant defense.

### Primary metabolite changes after exposure to Si and/or herbivory

**Figure 5:**
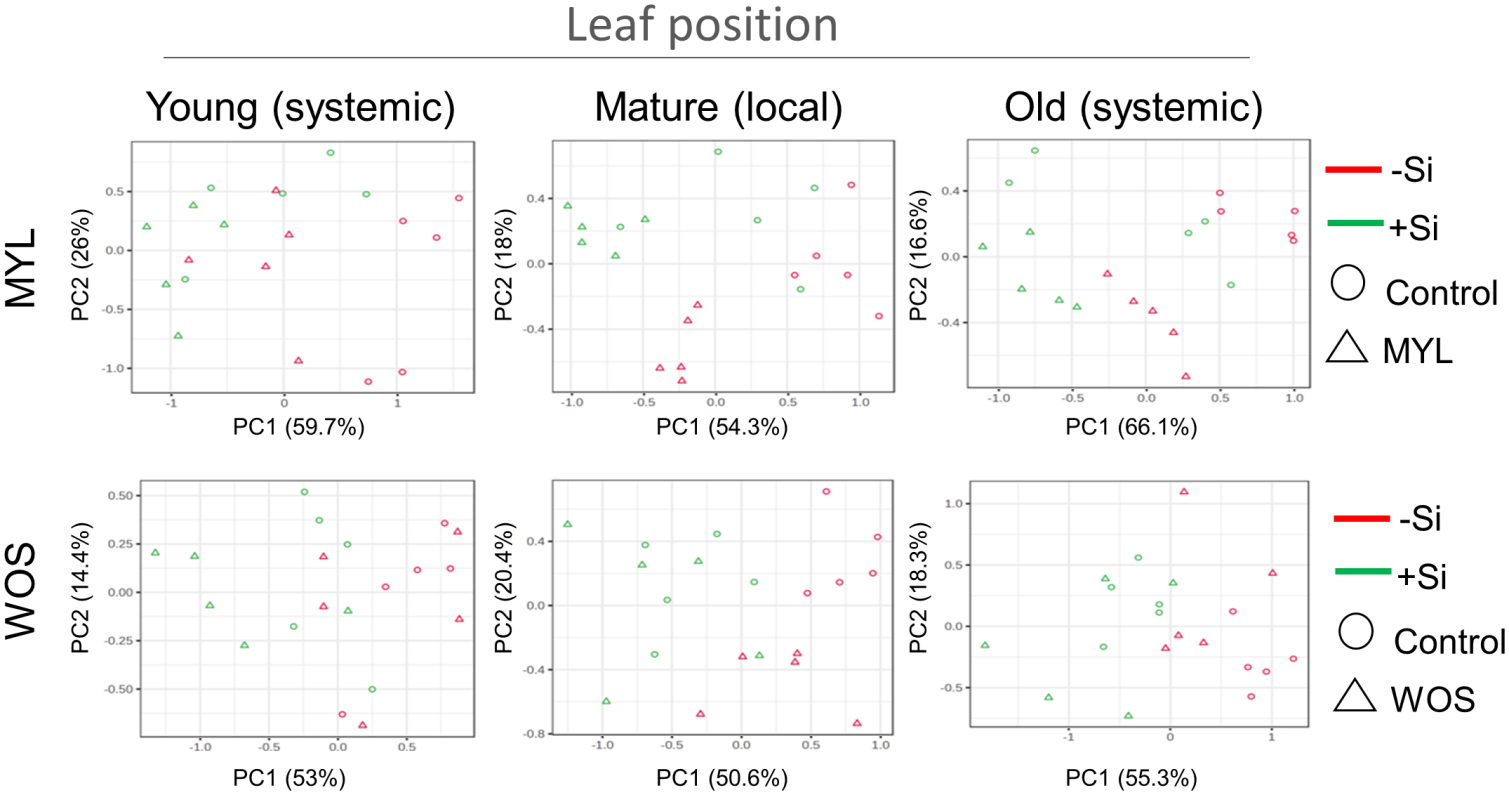
Primary metabolite changes after exposure of rice plants to Si alone or in combination with herbivory stress. Principal component analysis (PCA) of the primary metabolites accumulation in rice plants supplied with or without Si and subjected to either *M. loreyi* feeding (MYL) or elicitation with wounds treated with oral secretions from *M. loreyi* (WOS).

As Si-deprived rice plants grew significantly less in hydroponic media (**Figure 2B**), we hypothesized that Si supply may be altering the primary metabolic pathways in rice. We therefore performed an untargeted metabolomics screening by GC-MS, using N-methyl-N-trimethylsilyltrifluoroacetamide (MSTFA)-derivatized leaf extracts prepared from hydroponic rice plants. The plants kept for 7 weeks in the continuous presence or absence of Si, exposed to MYL feeding (or WOS), or left untreated for 24 hours, were used for analysis. The impact of Si and herbivory on primary metabolism was estimated from the levels of detectable sugars, amino acids, and organic acids reported in the **Supplementary Table 2**. The local MYL- or WOS-treated mature leaves (second leaf), one stage younger developing, and one stage older mature leaves were used to estimate the overall effect of Si and herbivory on the aboveground metabolism of rice. Using a principal component analysis (PCA) tool in MetaboAnalyst, although obtaining a fairly complex picture, certain patterns could be depicted. The samples from Si-cultivated plants generally tended to differ in PCA plots from those kept in Si-free media, and separated along the PC1 axis (**Figure 5**). Interestingly, samples from the MYL-exposed local leaves in Si-free media (red dots) were well separated from the controls, following PC2 axis direction, however, MYL-treated and control leaves from Si-supplemented media (green dots) partly overlapped (**Figure 5**). It suggests that MYL feeding differentially affects metabolic pathways in Si-dependent manner. Similar trends were observed in WOS-treated plants (**Figure 5**), although WOS effects were less pronounced compared to direct MYL feeding. Notably, direct MYL herbivory affected primary metabolic profiles in the older systemic leaves, while this was not the case in WOS treatments (**Figure 5**), suggesting that a stronger systemic signaling requires real feeding stimulus. Finally, we performed a Pearson’s correlation analysis implemented in MetaboAnalyst to estimate the importance of each primary metabolite in separation of samples after herbivory exposure and/or Si +/- supplementation in rice. While the accumulation of sugars was generally positively correlated with the presence of Si, multiple amino acids showed a negative correlation with Si (**Supplementary Figure 2A**). On the other hand, MYL treatment highlighted the importance of GABA as the main factor positively correlated with the exposure of rice to herbivory (**Supplementary Figure 2B**). GABA accumulation in Arabidopsis was previously shown to be induced by simulated herbivory as well as by real *S. littoralis* Boisduval feeding (Scholz et al., 2015).

In order to see if primary metabolic changes could be due to Si-dependent alteration of photosynthesis, we used a portable photosynthesis system (LI-COR) to determine the photosynthetic rates (Pn), transpiration rates (Tr), stomatal conductance (Gs), and intrinsic water use efficiency (iWUE) (**Supplementary Figure 3**). The results show that Pn was not influenced by Si, however, it decreased after WOS treatment, similar to Gs and Tr (**Supplementary Figure 3**). In addition, Gs significantly decreased in Si-supplemented plants treated with WOS, and as a result, iWUE was higher in these plants.

### Secondary metabolite levels in rice leaves exposed to Si and herbivory

**Figure 6:**
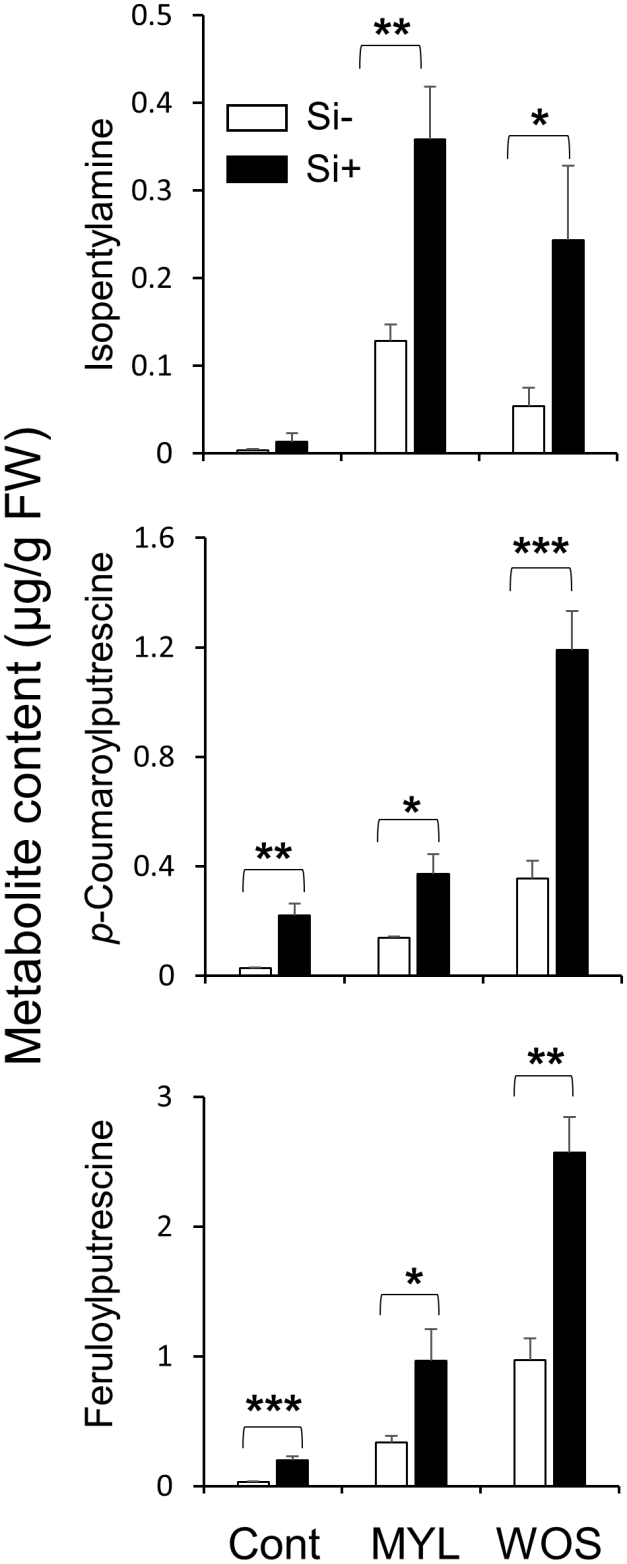
Effect of Si application on constitutive and induced levels of secondary metabolites in rice subjected to herbivory stress. Metabolite levels were determined with WT rice plants grown hydroponically with or without 0.5 mM Si for 7 weeks (Cont., Control) and then subjected to either *M. loreyi* larvae feeding (MYL) or wounds treated with oral secretions from *M. loreyi* (WOS) for 48 h. Data are means ± SE (n=5). Statistical differences within treatments (with and without Si) were analyzed by Student’s t-test (*P<0.05; **P<0.01; ***P<0.001; no symbol, not significant).

We next examined the accumulation of defense-related specialized (secondary) metabolites in Si-supplemented and Si-deprived rice exposed to either MYL infestation or WOS treatment. Si supply did not affect the constitutive levels of isopentylamine (IPA), but two phenolamides, N-*p*-coumaroylputrescine (CoP) and feruloylputrescine (FP) in rice leaves increased in some (but not all) experiments using Si-supplemented rice (**Figure 6**; **Supplementary Figure 4**). When exposed to MYL feeding for 48 h, Si-supplied rice leaves accumulated more IPA, CoP and FP relative to Si-deprived leaves (**Figure 6**; **Supplementary Figure 4**). Similarly, WOS treatment induced more IPA, CoP and FP in Si-supplied compared to Si-deprived rice leaves (**Figure 6**; **Supplementary Figure 4**). Overall, we detected more direct defense metabolites in Si-supplied leaves exposed to real or simulated herbivory stress, which was somewhat consistent with the more abundant hydrocarbon levels, such as sugars found in the Si-supplied rice leaves (**Supplementary Figure 2A**).

### Impact of Si on herbivore-induced plant volatile emissions in rice

**Figure 7:**
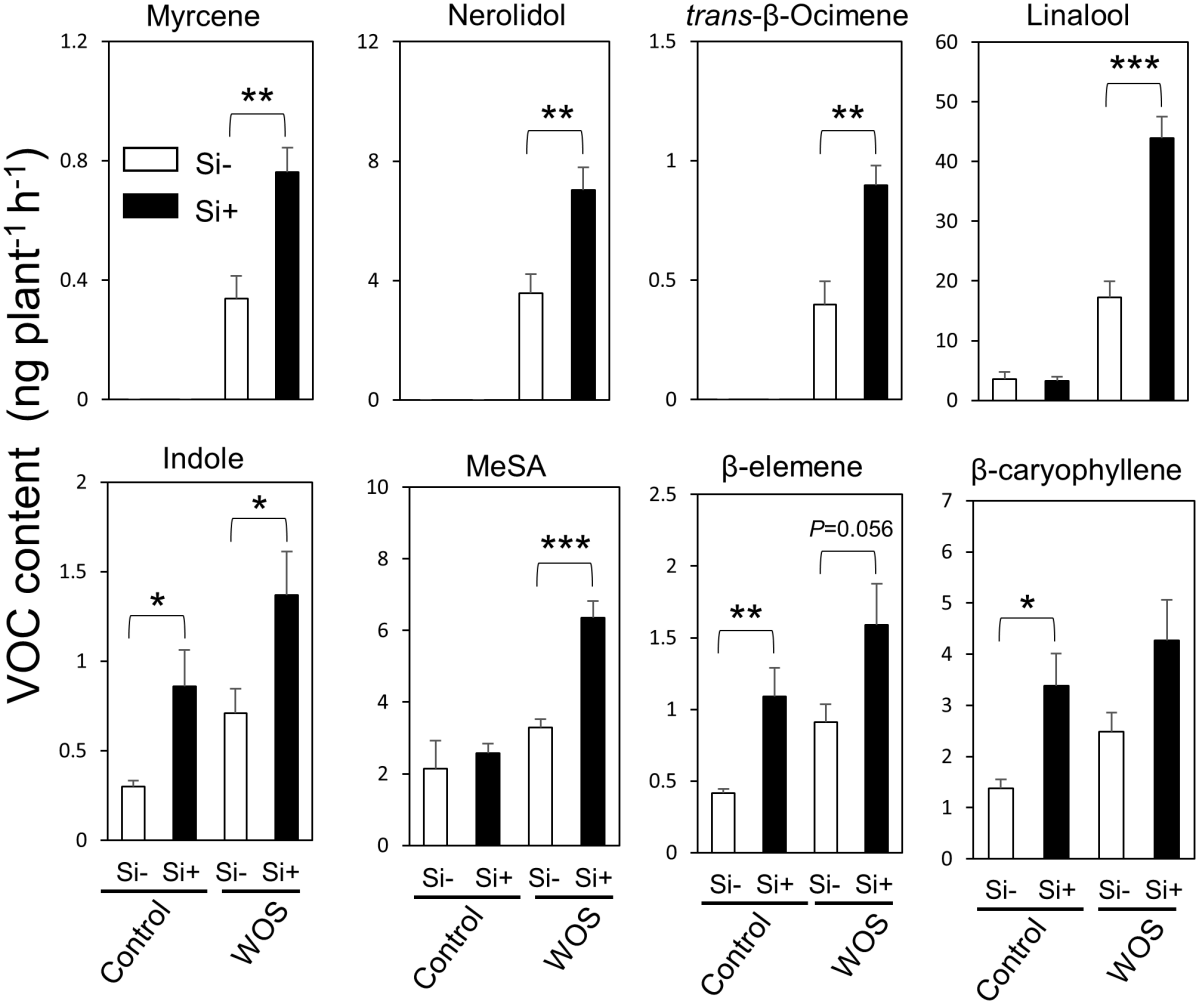
Si and WOS-regulated accumulation of VOCs emitted from rice leaves. Headspace volatile emissions from 8-weeks old WT rice plants grown hydroponically with or without 0.5 mM Si (Control), subjected to wounds treated with oral secretions from *M. loreyi* (WOS) for 24 h were collected and analyzed by GC-MS. WOS treatment was performed on two fully expanded leaves (youngest and mature). Data are mean relative amount ± SE (peak normalized to tetralin) (n=6). Statistical differences within treatments (Control and herbivory) were analyzed by Student’s t-test (*P<0.05; **P<0.01; ***P<0.001; no symbol, not significant).

In addition to direct defense, plants resist herbivores indirectly, i.e., through emissions of volatile organic compounds (VOCs) that attract natural enemies of herbivores. Taking into account the changes in primary and secondary metabolite levels after Si supplementation, we decided to test whether Si can also modify the emissions of VOCs in rice under herbivory stress. We collected and analyzed headspace VOCs from Si-supplied and Si-deprived plants after WOS treatment by GC-MS method. Constitutive levels of VOCs were not largely affected by Si presence, except for indole and sesquiterpenes, β-elemene and β-caryophyllene, which showed higher constitutive levels in Si-supplied compared to Si-deprived plants (**Figure 7**). After WOS treatment, Si-supplemented plants emitted higher quantities of numerous VOCs, including monoterpenes (myrcene, trans-β-ocimene, linalool), sesquiterpenes (nerolidol, β-elemene, β-caryophyllene), indole, and methyl salicylate, relative to Si-deprived plants (**Figure 7**). These data suggest that volatiles in rice headspace can be promoted by Si, which could be either because of the higher release and/or biosynthesis of these compounds. Subsequently, the expression of several volatile-related genes in rice was examined.

### Expression of terpene and phenylpropanoid-related genes

Stimulation of VOC release from rice is dependent on the increased expression of specific biosynthetic genes, such as *linalool synthase* (*LIS*) and *S-adenosyl methionine methyltransferase* (*SAMT*), involved in the rice linalool and methyl salicylate biosynthesis, respectively (Mujiono et al., 2020). When we measured the expression of *LIS*, induction of this gene was more profound in Si-containing plants relative to Si-deprived ones. However, this trend was not reflected in the expression of DXS3 located upstream in terpenoid biosynthetic pathway (**Supplementary Figure 5**). The transcript levels of *caryophyllene synthase* (*CAS*) were not induced by WOS as reported previously (Mujiono et al., 2020), and Si showed no consistent effect on the expression of this gene. The transcript levels of *phenylalanine ammonia lyase* (*PAL*) and *SAMT* for methyl salicylate were both elevated by Si, providing a support for the observed higher levels of MeSA in Si-supplemented rice headspace. In contrast to linalool and MeSA, increased levels of β-caryophyllene in rice headspace could not be explained by corresponding gene transcript data, suggesting that Si may be promoting release of β-caryophyllene by another mechanism.

### Impact of Si on JA accumulation in rice exposed to herbivory stress

**Figure 8:**
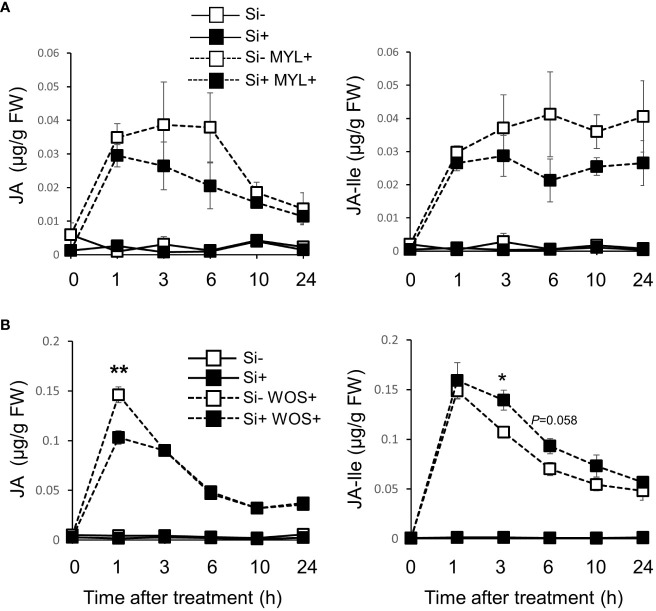
Si and herbivory-regulated accumulation of phytohormones in rice leaves. Jasmonic acid (JA) and jasmonoyl-L-isoleucine (JA-Ile) levels in mature rice leaves exposed to *M. loreyi* feeding (MYL; **A**) or in fully expanded young and mature rice leaves elicited with wounds treated with oral secretions from *M*. *loreyi* (WOS; **B**). Data are means ± SE (n=4). Statistical differences within pairs of MYL or WOS treatments were analyzed by Student’s t-test (*P<0.05; **P<0.01, no symbol, not significant).

Since jasmonic acid is a key factor in regulation of plant defense responses against insect herbivores, including specialized metabolism and VOC release, we examined the accumulation of JA and JA-Ile in Si-supplied and Si-deprived rice plants exposed to MYL infestation (**Figure 8A**). Si supplementation alone did not significantly alter the basal JA and JA-Ile levels in the observed 24 h period (**Figure 8A**). As expected, both hormones were strongly induced by MYL feeding, which surprisingly showed a more profound but non-significant trend in Si-deprived compared to Si-supplemented plants (**Figure 8A**). This trend was in contrary to our initial hypothesis that more specialized metabolites and VOCs could be due to elevation of defense signaling, based on the Si-mediated modulation of jasmonate levels. As we suspected that more feeding activity in Si-free plants (**Figure 1**) could be directly promoting jasmonates, we followed with the more standardized WOS treatment to test this possibility. Indeed, the levels of JA and JA-Ile were similar in most examined time points, except for 1h post elicitation, where JA was still higher in Si-deprived plants exposed to WOS (**Figure 8B**). These results generally rule out the possibility that higher metabolite levels in Si-supplemented plants could be due to higher levels of jasmonates after Si supplementation. In other hormones, abscisic acid (ABA) levels increased after MYL infestation and WOS treatment, however, Si supply did not cause any further changes (**Supplementary Figure 6**). Salicylic acid (SA) levels tended to be higher in Si-deprived media (**Supplementary Figure 6**).

### Ecological relevance of Si-regulated VOC releases

**Figure 9:**
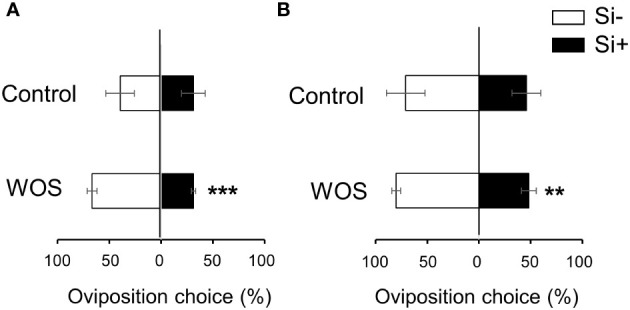
Effects of Si application on oviposition preference of gravid *M. loreyi* moths on rice plants. Oviposition choice of gravid *M. loreyi* moths (n=20) after 1 d **(A)** or 2 d **(B)** of release on WT rice plants grown hydroponically with or without 0.5 mM Si (Control) or subjected to wounds treated with oral secretions from *M*. *loreyi* (WOS) (n=12). WOS treatment was performed on fully expanded young and mature leaves. Data are means ± SE of four independent biological replicates. Statistical differences within pairs of treatments (with and without Si) were analyzed by Student’s t-test (**P<0.01; ***P<0.001; no symbol, not significant).

VOCs serve as danger as well as host-location cues for ovipositing females of herbivores (Achhami et al., 2021). We therefore investigated if differential emission of VOCs from Si-supplemented and Si-deprived rice plants, untreated or exposed to simulated herbivory, could change the oviposition behavior of MYL females. In cage choice-type experiments, Si alone did not affect the oviposition choice of MYL, even after 2 d of interaction with the plants (**Figures 9A, B**). However, in WOS-treated plants, more eggs were deposited on Si-deprived (67%) compared to Si-supplemented plants (31%) at 1 d after plant exposure to gravid moths (**Figure 9A**), which remained significant until two days (**Figure 9B**). Preference for Si-deprived rice plants treated with WOS, which generally released less volatiles (**Figure 7**), suggests that MYL females may be choosing a more suitable host plant based on the lower amount of volatiles, which may signal a lower defense capacity of the host to herbivores. On the other hand, silicon impregnation of leaves in control set of plants without herbivory did not seem to discourage MYL from laying eggs, although, presumably, the neonates would need to face a stronger feeding challenge after hatching from the eggs.

## Discussion

Uptake of silicon is essential for defense against chewing herbivores in numerous plant species (Liang et al., 2015). In addition to physical deterrence due to incorporation of Si into plant tissues, other possible mechanisms of plant resistance against insect herbivory regulated by Si remain unclear. In this report, we comprehensively examined the interactions between Si and defense traits in rice, which collectively revealed a potentially highly pleiotropic nature of Si involvement in plant-insect interactions.

### Si accumulation and rice defense against chewing herbivores

Rice accumulates up to 10% of Si in the shoots on dry weight basis, which is known to improve the grain yield, in addition to mitigating damages caused by environmental stresses (Ma, 2004; Tamai and Ma, 2008). In this study, Si supplementation increased the resistance of rice to larvae of MYL, which is a generalist chewing herbivore, relative to Si-deprived plants. However, Si-deprived rice supplied with Si 3 d before exposure to larvae could restore their resistance to the level comparable with the plants continuously grown in the Si-containing media (**Figure 1B**). This was consistent with the feeding performance of MYL larvae on cv. Nipponbare mutant *lsi1*, which is impaired in Si uptake (Suzuki et al., 2012), grown in the paddy field soil with normal Si levels in the laboratory (**Figure 1A**). Previously, extensive field damage was reported in similar *lsi1* mutants with cv. Oochikara genetic background in the field (Ma et al., 2006; Tamai and Ma, 2008). Interestingly, *lsi1* mutants tend to accumulate more lignin, possibly as a structural counter-response to gravity force (Suzuki et al., 2012), which could be also partially reverting the lack of structural toughness against herbivores in the Si-deprived plants.

The accumulation of Si was reported as part of the inducible defense arsenal in plants (Massey et al., 2007; Johnson et al., 2020). While the accumulation of Si differed with position (age) of rice leaves, directly insect-fed leaves contained significantly more Si compared to unfed controls (**Figures 2C**, **3A**). Si is acquired from soil as Si(OH)_4_ through the cooperation of specific influx and efflux root transporters, *Lsi1* and *Lsi2* (Ma et al., 2006; Ma et al., 2007b). Subsequently, Si is translocated to shoots by transpiration stream (Mitani et al., 2005), and then unloaded from xylem into leaf tissues by *Lsi6* (Yamaji et al., 2008). Recently, Mitani-Ueno et al. (2023) identified *SIET4* as a novel transporter required for deposition of Si on the rice leaf surface. Following these current updates, we found that the expression of *Lsi1* and *Lsi2* in the roots was only slightly altered by simulated herbivory (WOS) treatment (**Figure 4A**). In contrast, transcripts of *Lsi6* and *SIET4* were clearly upregulated in WOS-treated leaves at 1 h post elicitation (**Figure 4B**), suggesting that distribution and/or deposition rather than *de novo* uptake of Si by roots may be accounted for the higher levels of Si in herbivore attacked leaves. On the other hand, the expression of *Lsi1* in roots of rice infested by a specialist chewing herbivore, rice leaffolder (*Cnaphalocrocis medinalis* Guenée), was induced by approximately 2-fold (Lin et al., 2019), suggesting that stimulation of root Si uptake at transcriptional level may be dependent on herbivore species, or in particular, specialist vs. generalist features of the attackers.

### Impact of Si and herbivory stress on rice metabolic profiles

Earlier studies proposed that Si amendment alters basal gene expression in rice, which may be subsequently changing metabolic profiles, possibly as means of priming prior to and/or during stress responses (Brunings et al., 2009; Van Bockhaven et al., 2015; Frew et al., 2018; Jiang et al., 2022). In plant-pathogen interactions, application of Si to rice plants mediated differential expression of genes involved in nitrogen metabolism and transport, glycolysis and cell wall biosynthesis and degradation, while the amino acid metabolism-related genes were down-regulated (Van Bockhaven et al., 2015). In this study, we revealed a strong impact of Si on primary metabolites (e.g., soluble sugars, amino acids), which serve as structural and energy resources in plants, among others, used for the biosynthesis of specialized defense metabolites during herbivory stress (Wari et al., 2022). Soluble sugars tended to accumulate more in plants with Si, while amino acids content decreased with Si application (**Supplementary Figure 2A**). Against our assumption, leaf photosynthetic activity could not explain the observed Si-associated metabolic changes, such as higher sugar levels (**Supplementary Figure 3**). Nonetheless, it is possible that availability of sugars could, at least in part, contribute to increased growth rate of Si-supplemented relative to Si-deprived rice plants (**Figure 2B**). Previously, rice growth enhancement by Si under hydroponic conditions was reported by Lin et al. (2019) but particular mechanisms were not elucidated. Considering the significantly higher intrinsic water use efficiency (iWUE) in Si-supplemented plants subjected to herbivory stress (**Supplementary Figure 3**), it appears that Si amendment may positively affect the water usage in rice. Similar to our findings, Jiang et al. (2022) showed that Si application increased the iWUE of rice plants under drought stress.

Previous studies demonstrated that sugars in the absence of pathogens can trigger the expression of pathogenesis-related (PR) proteins (Moghaddam and Van den Ende, 2012; Rojas et al., 2014). In tobacco, leaf disks floated on sugars (glucose, fructose and sucrose) induced *PR* gene expression (Herbers et al., 1996). Thus, it appears that, in addition to higher Si accumulation, the increased levels of soluble sugars in Si-supplied rice may be partly preconditioning plants for a more efficient defense after herbivore attack. Interestingly, it was reported that reduced concentrations of Si and soluble sugars in *Lsi1* mutant rice affected resistance of mutants to *Bipolaris oryzae* (Breda de Haan) Shoemaker (Dallagnol et al., 2013). Furthermore, regardless of Si supply, we found a higher level of sugars in herbivore-infested plants relative to the controls (**Supplementary Figure 2B**, **Supplementary Table 2**). This is not surprising as plants have been reported to respond to herbivore attack by activating local catabolism of energy storage compounds like sucrose in order to recruit carbon sources for the production of defense-related metabolites (Zhou et al., 2015). In Arabidopsis, Ferrieri et al. (2012) showed that radioactively labeled carbohydrates were translocated to wounded or methyl-jasmonate-treated leaves, and used for the production of defense-related compounds such as phenolic glycosides and cinnamic acid. Moreover, the ability of *Populus* foliage to respond to herbivory or JA elicitation with elevated concentrations of phenolic compounds has been demonstrated to depend on the leaf’s ability to import carbohydrates from other plant modules (Arnold et al., 2004). Thus, the elevated accumulation of specialized defense metabolites in Si-supplied rice plants relative to the Si-deprived plants exposed to herbivory stress (**Figure 6**) could be, at least in part, due to a higher level of sugars in the rice leaves (**Supplementary Figures 2A, B**).

### Regulation of HIPVs in rice by Si

While energy metabolism and direct defenses are closely related, indirect plant defenses, which depend on volatile-mediated attraction of natural enemies of herbivores to attacked plants (Dudareva et al., 2006; Maffei et al., 2011), also demand a constant supply of plant energy. For instance, rice herbivore-induced plant volatiles (HIPVs) are mainly emitted during light photoperiod and strongly decline at night (Mujiono et al., 2021). Previously, plant HIPV emissions responded to Si supply (Liu et al., 2017; Leroy et al., 2019; de Oliveira et al., 2020), which is highly consistent with our current data (**Figure 7**). Si supplementation significantly elevated the headspace levels of several major HIPVs in rice, including linalool and methyl salicylate, compared to Si-deprived plants (**Figure 7**). However, it still remains unclear whether Si is altering the synthesis or emissions of HIPVs in rice.

To this end, we found an elevated level of transcripts of linalool synthase (*LIS*), phenylalanine ammonia lyase (*PAL*), and S-adenosyl methionine methyltransferase (*SAMT*) in WOS-challenged rice growing in the presence of Si, suggesting that Si may be promoting the emissions of linalool and methyl salicylate by amplifying their biosynthesis through transcriptional mechanisms (**Supplementary Figure 5**). Previously, defense genes including *CAT*, *SOD*, *PPO* and *POD* were also more expressed in Si-supplemented relative to Si-deprived rice infested with a rice leaffolder (Lin et al., 2019). As defense genes, such as *LIS* and *SAMT* are known to be controlled by jasmonic acid signaling upon wounding and herbivory, we purported the involvement of these hormones. However, a decreased level of jasmonates in Si-supplied plants under herbivory was found (**Figure 8**), which is not consistent with the more abundant transcripts of *LIS* and *SAMT*. In support of our data, Kim et al. (2014) also found that wounding stress resulted in higher JA accumulation in Si-deprived relative to Si-supplied rice plants. Similarly, in a conceptual model proposed for Si-JA relationship, Hall et al. (2019) suggested that Si-enriched plants generally show low levels of JA induction upon herbivore attack, possibly due to the available physical protection by Si. On the other hand, another study showed a higher expression of jasmonate biosynthesis genes (*LOX*, *AOS2*) in rice supplied with Si relative to Si-deprived plants (Lin et al., 2019), suggesting that these points need further clarifications.

Given the higher β-caryophyllene levels in headspace of rice grown in the presence of Si (**Figure 7**), that was not supported by transcriptional data (**Supplementary Figure 5**), it is likely that Si facilitates the release of volatiles by multiple independent mechanisms. For instance, plant cuticle may largely affect the release of volatiles from plants by passive diffusion mechanisms (Widhalm et al., 2015; Liao et al., 2021). As we did not examine the cuticle of rice leaves grown in the presence or absence of Si, it cannot be excluded that Si may be actually altering the cuticle thickness, similar to lignin deposition (Suzuki et al., 2012). Alternatively, Si could be altering the conductivity of apoplastic routes used for volatiles to escape from plants (Widhalm et al., 2015). The actual mechanisms involved in volatile production, release and gene expression changes mediated by Si remain as one of the most important tasks for future studies, fully acknowledging the important fact that volatiles and Si play crucial roles not only in defense but also in the host plant selection by ovipositing moth females (**Figure 9**).

## Data availability statement

The original contributions presented in the study are included in the article/**Supplementary Material**. Further inquiries can be directed to the corresponding author.

## Ethics statement

The manuscript presents research on animals that do not require ethical approval for their study.

## Author contributions

DO: Conceptualization, Data curation, Formal analysis, Investigation, Methodology, Validation, Visualization, Writing – original draft, Writing – review & editing. YH: Investigation, Methodology, Resources, Writing – review & editing, Writing – original draft. TS: Investigation, Methodology, Writing – review & editing, Conceptualization, Resources, Writing – original draft. NM: Conceptualization, Investigation, Methodology, Resources, Writing – review & editing, Data curation, Writing – original draft. IG: Conceptualization, Data curation, Funding acquisition, Investigation, Methodology, Project administration, Resources, Supervision, Writing – original draft, Writing – review & editing.

## Supplementary material

Supplementary Table 2 Primary metabolite changes after exposure of rice plants to Si alone or in combination with herbivory stress.

## References

[B1] Aboshi T. Iitsuka C. Galis I. Teraishi M. Kamo M. Nishimura A.. (2021). Isopentylamine is a novel defence compound induced by insect feeding in rice. Plant Cell Environ. 44, 247–256. doi: 10.1111/pce.13902 33034373

[B2] Achhami B. B. Reddy G. V. P. Hofland M. L. Sherman J. D. Peterson R. K. D. Weaver D. K. (2021). Plant volatiles and oviposition behavior in the selection of barley cultivars by wheat stem sawfly (Hymenoptera: Cephidae). Environ. Entomol. 50, 940–947. doi: 10.1093/ee/nvab035 33885745

[B3] Ahanger M. A. Bhat J. A. Siddiqui M. H. Rinklebe J. Ahmad P. (2020). Integration of silicon and secondary metabolites in plants: a significant association in stress tolerance. J. Exp. Bot. 71, 6758–6774. doi: 10.1093/jxb/eraa291 32585681

[B4] Alamgir K. M. Hojo Y. Christeller J. T. Fukumoto K. Isshiki R. Shinya T.. (2016). Systematic analysis of rice (*Oryza sativa*) metabolic responses to herbivory. Plant Cell Environ. 39, 453–466. doi: 10.1111/pce.12640 26386366

[B5] Alhousari F. Greger M. (2018). Silicon and mechanisms of plant resistance to insect pests. Plants (Basel) 7, 33. doi: 10.3390/plants7020033 29652790 PMC6027389

[B6] Aljbory Z. Chen M. (2018). Indirect plant defense against insect herbivores: a review. Insect Sci. 25, 2–23. doi: 10.1111/1744-7917.12436 28035791

[B7] Andama J. B. Mujiono K. Hojo Y. Shinya T. Galis I. (2020). Nonglandular silicified trichomes are essential for rice defense against chewing herbivores. Plant Cell Environ. 43, 2019–2032. doi: 10.1111/pce.13775 32323332

[B8] Arnold T. Appel H. Patel V. Stocum E. Kavalier A. Schultz J. (2004). Carbohydrate translocation determines the phenolic content of Populus foliage: a test of the sink-source model of plant defense. New Phytol. 164, 157–164. doi: 10.1111/j.1469-8137.2004.01157.x 33873480

[B9] Balakrishnan D. Bateman N. Kariyat R. R. (2024). Rice physical defenses and their role against herbivores. Planta 259, 110. doi: 10.1007/s00425-024-04381-7 38565704 PMC10987372

[B10] Brunings A. M. Datnoff L. E. Ma J. F. Mitani N. Nagamura Y. Rathinasabapathi B.. (2009). Differential gene expression of rice in response to silicon and rice blast fungus *Magnaporthe oryzae* . Ann. Appl. Biol. 155, 161–170. doi: 10.1111/j.1744-7348.2009.00347.x

[B11] Dallagnol L. J. Rodrigues F. A. Chaves A. R. M. Vale F. X. R. DaMatta F. M. (2013). Photosynthesis and sugar concentration are impaired by the defective active silicon uptake in rice plants infected with *Bipolaris oryzae* . Plant Pathol. 62, 120–129. doi: 10.1111/j.1365-3059.2012.02606.x

[B12] Debona D. Rodrigues F. A. Datnoff L. E. (2017). Silicon’s role in abiotic and biotic plant stresses. Annu. Rev. Phytopathol. 55, 85–107. doi: 10.1146/annurev-phyto-080516-035312 28504920

[B13] de Oliveira R. S. Penaflor M, F, G, V. Goncalves F. G. Sampaio M. V. Korndorfer A. P. Silva W. D.. (2020). Silicon-induced changes in plant volatiles reduce attractiveness of wheat to the bird-cherry-oat aphid *Rhopalosiphum padi* and attract the parasitoid *Lysiphlebus testaceipes* . PloS One 15, e0231005. doi: 10.1371/journal.pone.0231005 32243466 PMC7122784

[B14] Detmann K. C. Araujo W. L. Martins S. C. V. Sanglard L. M. V. P. Reis J. V. Detmann E.. (2012). Silicon nutrition increases grain yield, which, in turn, exerts a feed-forward stimulation of photosynthetic rates via enhanced mesophyll conductance and alters primary metabolism in rice. New Phytol. 196, 752–762. doi: 10.1111/j.1469-8137.2012.04299.x 22994889

[B15] dos Santos M. S. Sanglard L. M. P. V. Martins S. C. V. Barbosa M. L. de Melo D. C. Gonzaga W. F.. (2019). Silicon alleviates the impairments of iron toxicity on the rice photosynthetic performance via alterations in leaf diffusive conductance with minimal impacts on carbon metabolism. Plant Physiol. Biochem. 143, 275–285. doi: 10.1016/j.plaphy.2019.09.011 31536896

[B16] Dudareva N. Negre F. Nagegowda D. A. Orlova I. (2006). Plant volatiles: recent advances and future perspectives. Crit. Rev. Plant Sci. 25, 417–440. doi: 10.1080/07352680600899973

[B17] Fautex F. Chain F. Belzile F. Menzies J. G. Belanger R. R. (2006). The protective role of silicon in the Arabidopsis-powdery mildew pathosystem. Proc. Natl. Acad. Sci. U.S.A. 103, 17554–17559. doi: 10.1073/pnas.0606330103 17082308 PMC1859967

[B18] Fawe A. Abou-Zaid M. Menzies J. G. Belanger R. R. (1998). Silicon-mediated accumulation of flavonoid phytoalexins in cucumber. Phytopathol. 88, 396–401. doi: 10.1094/PHYTO.1998.88.5.396 18944917

[B19] Ferrieri A. P. Appel H. Ferrieri R. A. Schultz J. C. (2012). Novel application of 2- [(18)F] fluoro-2-deoxy-D-glucose to study plant defenses. Nucl. Med. Biol. 39, 1152–1160. doi: 10.1016/j.nucmedbio.2012.06.005 22795788

[B20] Frew A. Weston L. A. Reynolds O. L. Gurr G. M. (2018). The role of silicon in plant biology: a paradigm shift in research approach. Ann. Bot. 121, 1265–1273. doi: 10.1093/aob/mcy009 29438453 PMC6007437

[B21] Fukumoto K. Alamgir K. Yamashita Y. Mori I. C. Matsuura H. Galis I. (2013). Response of rice to insect elicitors and the role of OsJAR1 in wound and herbivory-induced JA-Ile accumulation. J. Integr. Plant Biol. 55, 775–784. doi: 10.1111/jipb.12057 23621526

[B22] Gulzar A. Wright D. J. (2015). Sub-lethal effects of Vip3A toxin on survival, development and fecundity of *Heliothis virescens* and *Plutella xylostella* . Ecotoxicology 24, 1815–1822. doi: 10.1007/s10646-015-1517-6 26162322

[B23] Hajiboland R. Cheraghvareh L. Poschenrieder C. (2017). Improvement of drought tolerance in tobacco (*Nicotiana rustica* L.) plants by silicon. J. Plant Nutr. 40, 1661–1676. doi: 10.1080/01904167.2017.1310887

[B24] Hall C. R. Waterman J. M. Vandegeer R. K. Hartley S. E. Johnson S. N. (2019). The role of silicon in antiherbivore phytohormonal signalling. Front. Plant Sci. 10, 1132. doi: 10.3389/fpls.2019.01132 31620157 PMC6759751

[B25] Han Y. Li P. Gong S. Yang L. Wen L. Hou M. (2016). Defense responses in rice induced by silicon amendment against infestation by the leaf folder *Cnaphalocrocis medinalis* . PloS One 11, e0153918. doi: 10.1371/journal.pone.0153918 27124300 PMC4849577

[B26] Hanley M. E. Lamont B. B. Fairbanks M. M. Rafferty C. M. (2007). Plant structural traits and their role in anti-herbivore defence. Perspec. Plant Ecol. Evol. Syst. 8, 157–178. doi: 10.1016/j.ppees.2007.01.001

[B27] Herbers K. Meuwly P. Metraux J. P. Sonnewald U. (1996). Salicylic acid-independent induction of pathogenesis-related protein transcripts by sugars is dependent on leaf developmental stage. FEBS Lett. 397, 239–244. doi: 10.1016/S0014-5793(96)01183-0 8955355

[B28] Jiang H. Song Z. Su Q. Wei Z. Li W. Jiang Z.. (2022). Transcriptomic and metabolomic reveals silicon enhances adaptation of rice under dry cultivation by improving flavonoid biosynthesis, osmoregulation, and photosynthesis. Front. Plant Sci. 13, 967537. doi: 10.3389/fpls.2022.967537 35991391 PMC9386530

[B29] Johnson S. N. Rowe R. C. Hall C. R. (2020). Silicon is an inducible and effective herbivore defence against *Helicoverpa punctigera* (Lepidoptera: Noctuidae) in soybean. Bull. Entomol. Res. 110, 417–422. doi: 10.1017/S0007485319000798 31813402

[B30] Kaur I. Kariyat R. (2023). Trichomes mediate plant-herbivore interactions in two Cucurbitaceae species through pre- and post-ingestive ways. J. Pest Sci. 96, 1077–1089. doi: 10.1007/s10340-023-01611-x PMC10047472 37168103

[B31] Kim Y. Khan A. L. Waqas M. Jeong H. Kim D. Shin J. S.. (2014). Regulation of jasmonic acid biosynthesis by silicon application during physical injury to *Oryza sativa* L. J. Plant Res. 127, 525–532. doi: 10.1007/s10265-014-0641-3 24840865

[B32] Konno K. Inoue T. A. Nakamura M. (2014). Synergistic defensive function of raphides and protease through the needle effect. PloS One 9, e91341. doi: 10.1371/journal.pone.0091341 24621613 PMC3951349

[B33] Korth K. L. Doege S. J. Park S. H. Goggin F. L. Wang Q. Gomez S. K.. (2006). *Medicago truncatula* mutants demonstrate the role of plant calcium oxalate crystals as an effective defense against chewing insects. Plant Physiol. 141, 188–195. doi: 10.1104/pp.106.076737 16514014 PMC1459329

[B34] Leroy N. de Tombeur F. Walgraffe Y. Cornelis J. Verheggen F. J. (2019). Silicon and plant natural defenses against insect pests: impact on plant volatile organic compounds and cascade effects on multitrophic interactions. Plants (Basel) 8, 444. doi: 10.3390/plants8110444 31652861 PMC6918431

[B35] Leroy N. Martin C. Arias A. A. Cornelis J. Verheggen F. J. (2022). If all else fails: impact of silicon accumulation in maize leaves on volatile emissions and oviposition site selection of *Spodoptera exigua* Hubner. J. Chem. Ecol. 48, 841–849. doi: 10.1007/s10886-022-01386-y 36302913

[B36] Liang Y. Nikolic M. Belanger R. Gong H. Song A. (2015). Silicon in Agriculture (Dordrecht: Springer). doi: 10.1007/978-94-017-9978-2

[B37] Liao P. Ray S. Boachon B. Lynch J. H. Deshpande A. McAdam S.. (2021). Cuticle thickness affects dynamics of volatile emission from petunia flowers. Nat. Chem. Biol. 17, 138–145. doi: 10.1038/s41589-020-00670-w 33077978

[B38] Lin Y. Sun Z. Li Z. Xue R. Cui W. Sun S.. (2019). Deficiency in silicon transporter Lsi1 compromises inducibility of anti-herbivore defense in rice plants. Front. Plant Sci. 10, 652. doi: 10.3389/fpls.2019.00652 31178878 PMC6543919

[B39] Liu J. Zhu J. Zhang P. Han L. Reynolds O. L. Zeng R.. (2017). Silicon supplementation alters the composition of herbivore induced plant volatiles and enhances attraction of parasitoids to infested rice plants. Front. Plant Sci. 8, 1265. doi: 10.3389/fpls.2017.01265 28769965 PMC5515826

[B40] Ma J. F. (2004). Role of silicon in enhancing the resistance of plants to biotic and abiotic stresses. Soil Sci. Plant Nutr. 50, 11–18. doi: 10.1080/00380768.2004.10408447

[B41] Ma J. F. Tamai K. Ichii M. Wu G. F. (2002). A rice mutant defective in Si uptake. Plant Physiol. 130, 111–117. doi: 10.1104/pp.010348 12481095 PMC166723

[B42] Ma J. F. Tamai K. Yamaji N. Mitani N. Konishi S. Katsuhara M.. (2006). A silicon transporter in rice. Nature. 440, 688–691. doi: 10.1038/nature04590 16572174

[B43] Ma J. F. Yamaji N. Mitani N. Tamai K. Konishi S. Fujiwara T.. (2007b). An efflux transporter of silicon in rice. Nature. 448, 209–212. doi: 10.1038/nature05964 17625566

[B44] Ma J. F. Yamaji N. Tamai K. Mitani N. (2007a). Genotypic difference in silicon uptake and expression of silicon transporter genes in rice. Plant Physiol. 145, 919–924. doi: 10.1104/pp.107.107599 17905867 PMC2048782

[B45] Maffei M. E. Gertsch J. Appendino G. (2011). Plant volatiles: production, function and pharmacology. Nat. Prod. Rep. 28, 1359–1380. doi: 10.1039/c1np00021g 21670801

[B46] Massey F. P. Ennos A. R. Hartley S. E. (2007). Herbivore specific induction of silica-based plant defences. Oecologia. 152, 677–683. doi: 10.1007/s00442-007-0703-5 17375331

[B47] McMillan H. N. (2023). Plant target gut microbes to reduce insect herbivore damage. Proc. Natl. Acad. Sci. U.S.A. 120, e2308568120. doi: 10.1073/pnas.2308568120 37379352 PMC10334768

[B48] Mitani N. Ma J. F. Iwashita T. (2005). Identification of the silicon form in xylem sap of rice (Oryza sativa L.). Plant Cell Physiol. 46, 279–283. doi: 10.1093/pcp/pci018 15695469

[B49] Mitani-Ueno N. Yamaji N. Huang S. Yoshioka Y. Miyaji T. Ma J. F. (2023). A silicon transporter gene required for healthy growth of rice on land. Nat. Commun. 14, 6522. doi: 10.1038/s41467-023-42180-y 37857615 PMC10587147

[B50] Mitchell C. Brennan R. M. Graham J. Karley A. J. (2016). Plant defense against herbivores pests: exploiting resistance and tolerance traits for sustainable crop protection. Front. Plant Sci. 7, 1132. doi: 10.3389/fpls.2016.01132 27524994 PMC4965446

[B51] Moghaddam M. R. B. Van den Ende W. (2012). Sugars and plant innate immunity. J. Exp. Bot. 63, 3989–3998. doi: 10.1093/jxb/ers129 22553288

[B52] Mujiono K. Tohi T. Sobhy I. S. Hojo Y. Ho N. T. Shinya T.. (2020). Ethylene functions as a suppressor of volatile production in rice. J. Exp. Bot. 71, 6491–6511. doi: 10.1093/jxb/eraa341 32697299

[B53] Mujiono K. Tohi T. Sobhy I.S. Hojo Y. Shinya T. Galis I. (2021). Herbivore-induced and constitutive volatiles are controlled by different oxylipin-dependent mechanisms in rice. Plant Cell Environ. 44, 2687–2699. doi: 10.1111/pce.14126 34114241

[B54] Pereira P. Nascimento A. M. de Souza B. H. S. Peñaflor M. F. G. V. (2021). Silicon supplementation of maize impacts Fall Armyworm colonization and increases predator attraction. Neotrop. Entomol. 50, 654–661. doi: 10.1007/s13744-021-00891-1 34184235

[B55] Raviv B. Khadka J. Swetha B. Singiri J. R. Grandhi R. Shapira E.. (2020). Extreme drought alters progeny dispersal unit properties of winter wild oat (*Avena sterilis* L.). Planta. 252, 77. doi: 10.1007/s00425-020-03491-2 33033936

[B56] R Core Team (2023). R: a Language and Environment for Statistical Computing (Vienna, Austria). Available at: https://cran.ism.ac.jp/.

[B57] Reynolds O. L. Padula M. P. Zeng R. Gurr G. M. (2016). Silicon: potential to promote direct and indirect effects on plant defense against arthropods pests in agriculture. Front. Plant Sci. 7, 744. doi: 10.3389/fpls.2016.00744 27379104 PMC4904004

[B58] Rojas C. M. Senthil-Kumar M. Tzin V. Mysore K. S. (2014). Regulation of primary plant metabolism during plant-pathogen interactions and its contribution to plant defense. Front. Plant Sci. 5, 17. doi: 10.3389/fpls.2014.00017 24575102 PMC3919437

[B59] Scholz S. S. Reichelt M. Mekonnen D. W. Ludewig F. Mithöfer A. (2015). Insect herbivory-elicited GABA accumulation in plants is a wound-induced, direct, systemic, and jasmonate-independent defense response. Front. Plant Sci. 6, 1128. doi: 10.3389/fpls.2015.01128 26734035 PMC4686679

[B60] Schuman M. C. Baldwin I. T. (2016). The layers of plant responses to insect herbivores. Annu. Rev. Entomol. 61, 373–394. doi: 10.1146/annurev-ento-010715-023851 26651543

[B61] Shinya T. Hojo Y. Desaki Y. Christeller J. T. Okada K. Shibuya N.. (2016). Modulation of plant defense responses to herbivores by simultaneous recognition of different herbivore-associated elicitors in rice. Sci. Rep. 6, 32537. doi: 10.1038/srep32537 27581373 PMC5007475

[B62] Sobhy I. S. Miyake A. Shinya T. Galis I. (2017). Oral secretions affect HIPVs induced by generalist (*Mythimna loreyi*) and specialist (*Parnara guttata*) herbivores in rice. J. Chem. Ecol. 43, 929–943. doi: 10.1007/s10886-017-0882-4 28861807

[B63] Suzuki S. Ma J. F. Yamamoto N. Hattori T. Sakamoto M. Umezawa T. (2012). Silicon deficiency promotes lignin accumulation in rice. Plant Biotechnol. 29, 391–394. doi: 10.5511/plantbiotechnology.12.0416a

[B64] Tamai K. Ma J. F. (2008). Reexamination of silicon effects on rice growth and production under field conditions using a low silicon mutant. Plant Soil. 307, 21–27. doi: 10.1007/s11104-008-9571-y

[B65] Tanabe K. Hojo Y. Shinya T. Galis I. (2016). Molecular evidence for biochemical diversification of phenolamide biosynthesis in rice plants. J. Integr. Plant Biol. 58, 903–913. doi: 10.1111/jipb.12480 27015846

[B66] Van Bockhaven J. Steppe K. Bauweraerts I. Kikuchi S. Asano T. Hofte M.. (2015). Primary metabolism plays a central role in moulding silicon-inducible brown spot resistance in rice. Mol. Plant Pathol. 16, 811–824. doi: 10.1111/mpp.12236 25583155 PMC6638399

[B67] Wari D. Aboshi T. Shinya T. Galis I. (2022). Integrated view of plant metabolic defense with particular focus on chewing herbivores. J. Integr. Plant Biol. 64, 449–475. doi: 10.1111/jipb.13204 34914192

[B68] Wei-min D. Ke-qin Z. Bin-wu D. Cheng-xiao S. Kang-le Z. Run C.. (2005). Rapid determination of silicon content in rice. Rice Sci. 12, 145–147.

[B69] Widhalm J. R. Jaini R. Morgan J. A. Dudareva N. (2015). Rethinking how volatiles are released from plant cells. Trends Plant Sci. 20, 545–550. doi: 10.1016/j.tplants.2015.06.009 26189793

[B70] Yamaji N. Mitatni N. Ma J. F. (2008). A transporter regulating silicon distribution in rice shoots. Plant Cell. 20, 1381–1389. doi: 10.1105/tpc.108.059311 18515498 PMC2438455

[B71] Ye M. Song Y. Long J. Wang R. Baerson S. R. Pan Z.. (2013). Priming of jasmonate-mediated antiherbivore defense responses in rice by silicon. Proc. Natl. Acad. Sci. U.S.A. 110, E3631–E3639. doi: 10.1073/pnas.1305848110 24003150 PMC3780902

[B72] Zakir A. Sadek M. M. Bengtsson M. Hansson B. S. Witzgall P. Anderson P. (2013). Herbivore-induced plant volatiles provide associational resistance against an ovipositing herbivore. J. Ecol. 101, 410–417. doi: 10.1111/1365-2745.12041

[B73] Zhou S. Jander G. (2022). Molecular ecology of plant volatiles in interactions with insect herbivores. J. Exp. Bot. 73, 449–462. doi: 10.1093/jxb/erab413 34581787

[B74] Zhou S. Lou Y. Tzin V. Jander G. (2015). Alteration of plant primary metabolism in response to insect herbivory. Plant Physiol. 169, 1488–1498. doi: 10.1104/pp.15.01405 26378101 PMC4634104

