# Supplementary material for: Comprehensive analysis of silicon impact on defense and metabolic responses in rice exposed to herbivory stress
Source: Front Plant Sci. 2024 May 30;15:1399562. doi: 10.3389/fpls.2024.1399562 (PMC11169889; doi:10.3389/fpls.2024.1399562)
Supplement: Supplementary file 1 [file Table_1.docx]

**Supplementary Table 1**

Oligonucleotide primers used in the study

**RAP-DB** **Forward primer (5’ -> 3’)**   **Reverse primer (5’ >3’)**

| *LOW SILICON RICE 1 (OsLsi1)* | *Os02g0745100* | GCCAGCAACAACTCGAGAACAA | CATGGTAGGCATGGTGCCGT |
| --- | --- | --- | --- |
| *LOW SILICON RICE 2 (OsLsi2)* | *Os03g0107300* | ATCACCTTCCCCAAGTTCCT | CAGCTCCCTCCAGTACATGC |
| *LOW SILICON RICE 6 (OsLsi6)* | *Os06g0228200* | ACATGATGTTCGTCACCTGC | GAAGTAGATCCAGAGGCCGG |
| *SILICON EFFLUX TRANSPORTER 4 (OsSIET4)* | *Os03g0147400* | AAGCAGACGGTGATTGAGAAGG | GCATGTGCAGTTGTACAAACACC |
| *1-DEOXY-D-XYLULOSE 5-PHOSPHATE SYNTHASE 3 (OsDXS3)* | *Os07g0190000* | GGGGGAGGTTCCAGTAAGAA | TCATTTTGCATTTGGAAGCA |
| *LINALOOL SYNTHASE (OsLIS)* | *Os02g0121700* | CCAGGATGGTCGGCGTCATG | CACGCCATTATGCATGGACGATG |
| *CARYOPHYLLENE SYNTHASE (OsCAS)* | *Os08g0139700* | ATCGCCGGGGAGTGTCTC | GGAGTGTATTGTATCCTTGAGCG |
| *PHENYLALANINE AMMONIA-LYASE (OsPAL)* | *Os02g0626600* | CTACCCGCTGATGAAGAAGC | CTACCCGCTGATGAAGAAGC |
| *S-ADENOSYL-L-METHIONINE: SALICYLIC ACID CARBOXYL METHYLTRANSFERASE (OsSAMT)* | *Os02g0719600* | CTCATCGCTCGTCATTTCGG | ACACACTGGCACGCTACTTA |
