# Supplementary material for: Comprehensive analysis of silicon impact on defense and metabolic responses in rice exposed to herbivory stress
Source: Front Plant Sci. 2024 May 30;15:1399562. doi: 10.3389/fpls.2024.1399562 (PMC11169889; doi:10.3389/fpls.2024.1399562)
Supplement: Supplementary file 3 [file DataSheet_1.pdf]

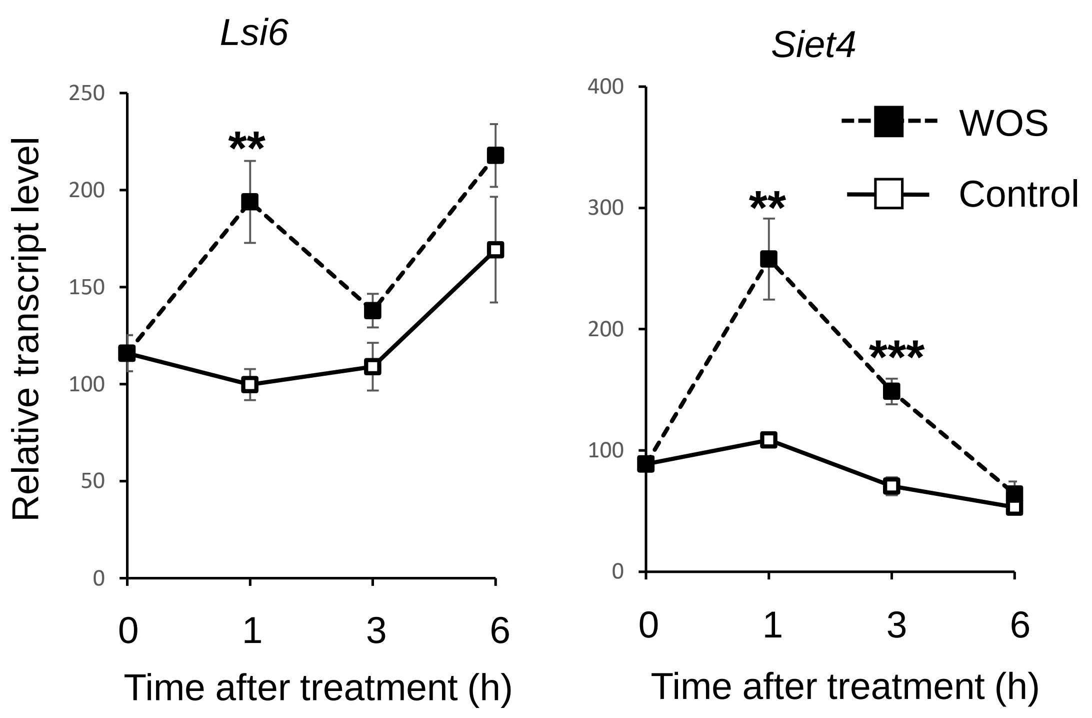


**Supplementary Figure 1**. **Transcript levels of *Lsi6* and *SIET4* in WT rice plants after exposure to herbivory stress.** Gene expression was determined in fully expanded rice leaves (young and mature leaves) at indicated time intervals by qRT-PCR in 7-week-old WT rice plants before and after elicitation with wounds treated with oral secretions from *M. loreyi* (WOS). Data are mean ± SE (n=4). Statistical differences between treatments (Control and WOS) were analyzed by Student’s t-test (**P<0.01; ***P<0.001; no symbol, not significant).

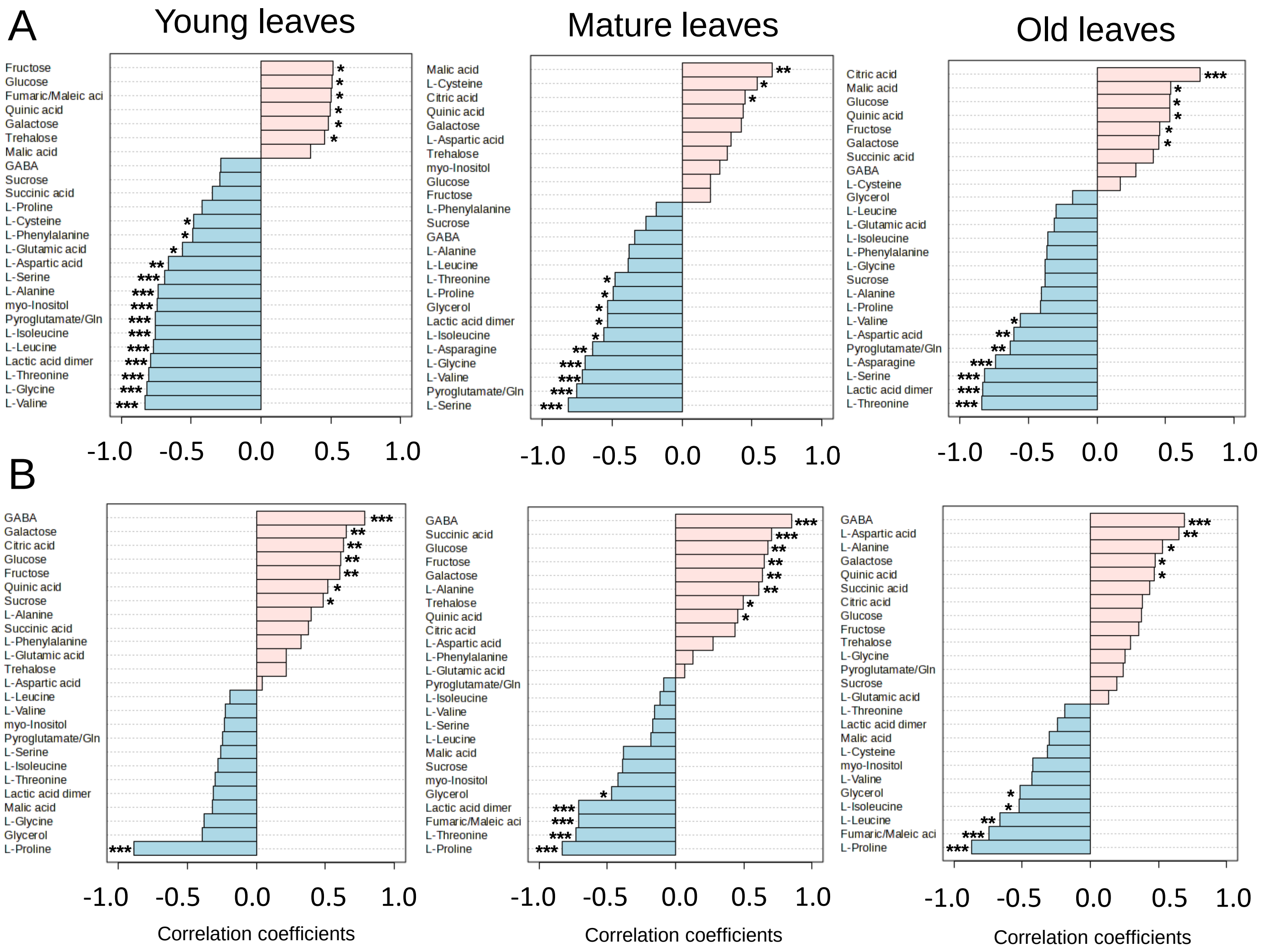


**Supplementary Figure 2. Primary metabolite changes after exposure of rice plants to Si alone or in combination with herbivory stress.** Correlation plot showing association between primary metabolites and (A) Si amendment or (B) *M. loreyi* (MYL) herbivory treatment in rice leaves. Asterisks indicate p-values (*P<0.05; **P<0.01; ***P<0.001; no symbol, not significant).

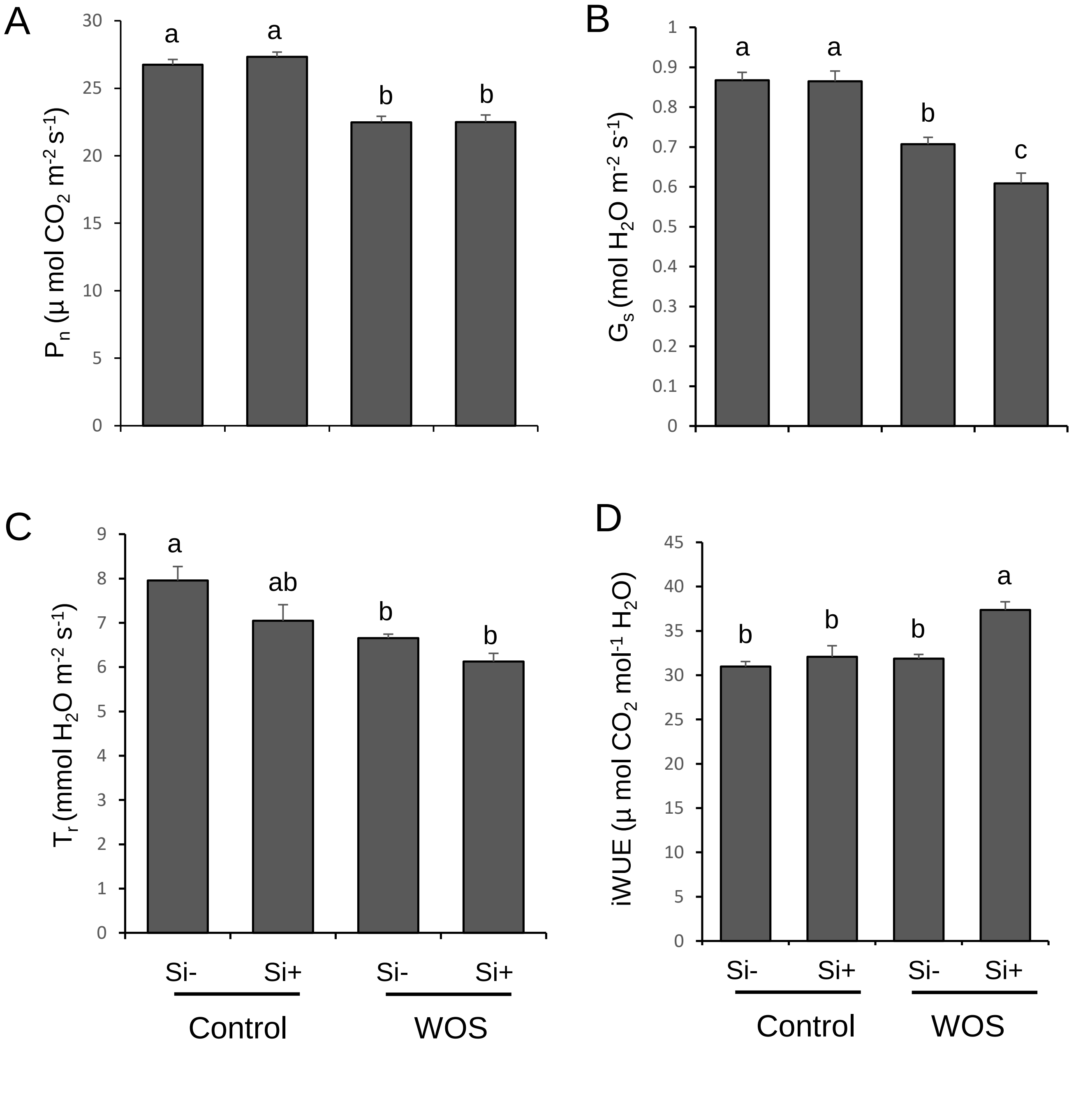


**Supplementary Figure 3. Impact of Si on leaf photosynthetic parameters of rice exposed to herbivory stress.** (A) Net photosynthetic rates, Pn; (B) Stomatal conductivity, Gs; (C) Transpiration rate, Tr; (D) Intrinsic water-use efficiency, iWUE. Photosynthetic parameters were measured in mature leaves of 7-week-old WT rice plants before and after elicitation with wounds treated with oral secretions from *M. loreyi* (WOS). Data are means ± SE (n=5 for Si treatments, n=4 for WOS treatments). Data followed by different letters (a-c) indicate significant difference ($P \leq 0.05$) according to ANOVA followed by Tukey's HSD test.

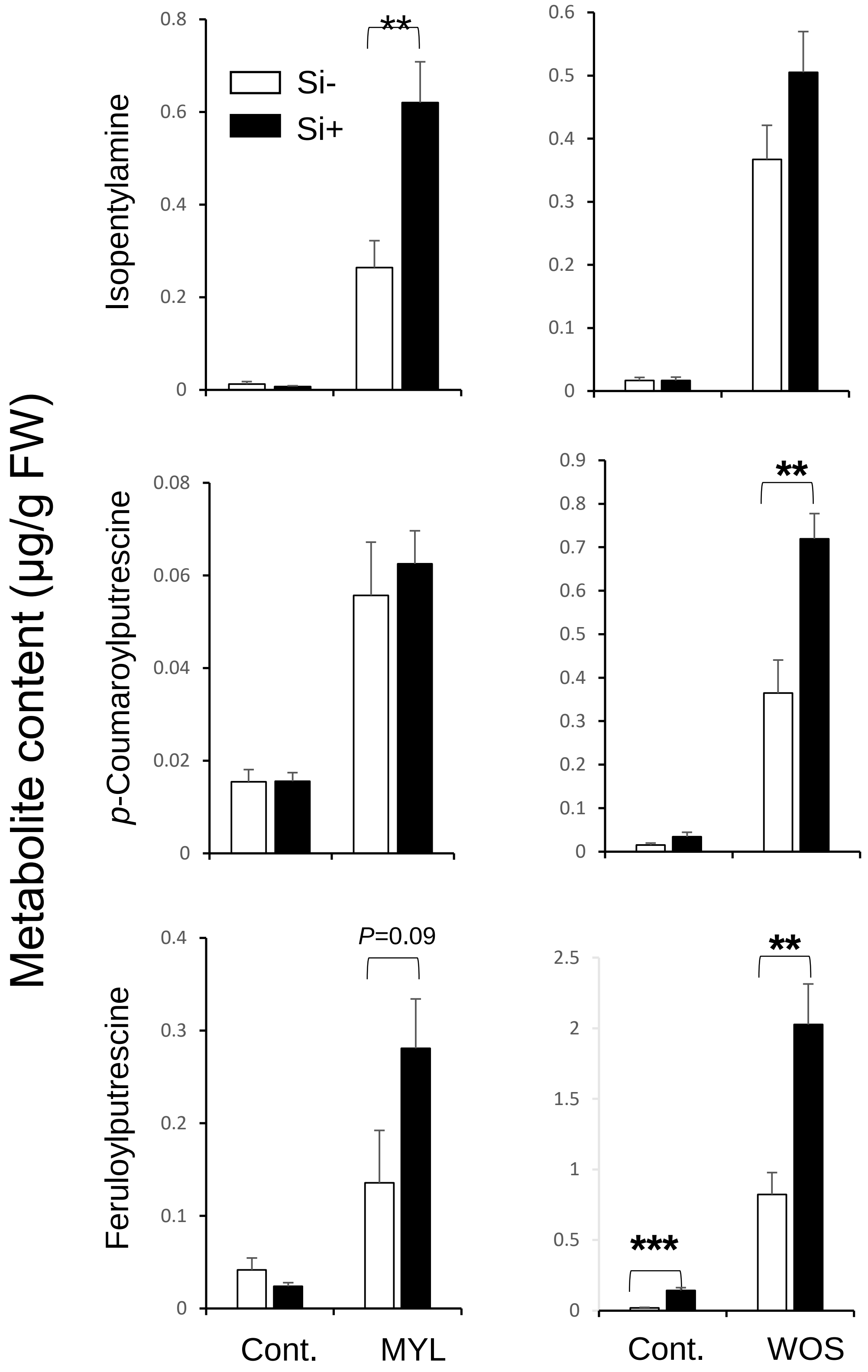


**Supplementary Figure 4. Effect of Si application on constitutive and induced levels of secondary metabolites in rice subjected to herbivory stress.** Metabolite levels were determined with WT rice plants grown hydroponically with or without 0.5 mM Si for 7 weeks (Cont., Control) and then subjected to either *M. loreyi* larvae feeding (MYL) or wounds treated with oral secretions from *M. loreyi* (WOS) for 48 h. Data are means ± SE (n=5). Statistical differences within treatments (with and without Si) were analyzed by Student's t-test (**P<0.01; ***P<0.001; no symbol, not significant).

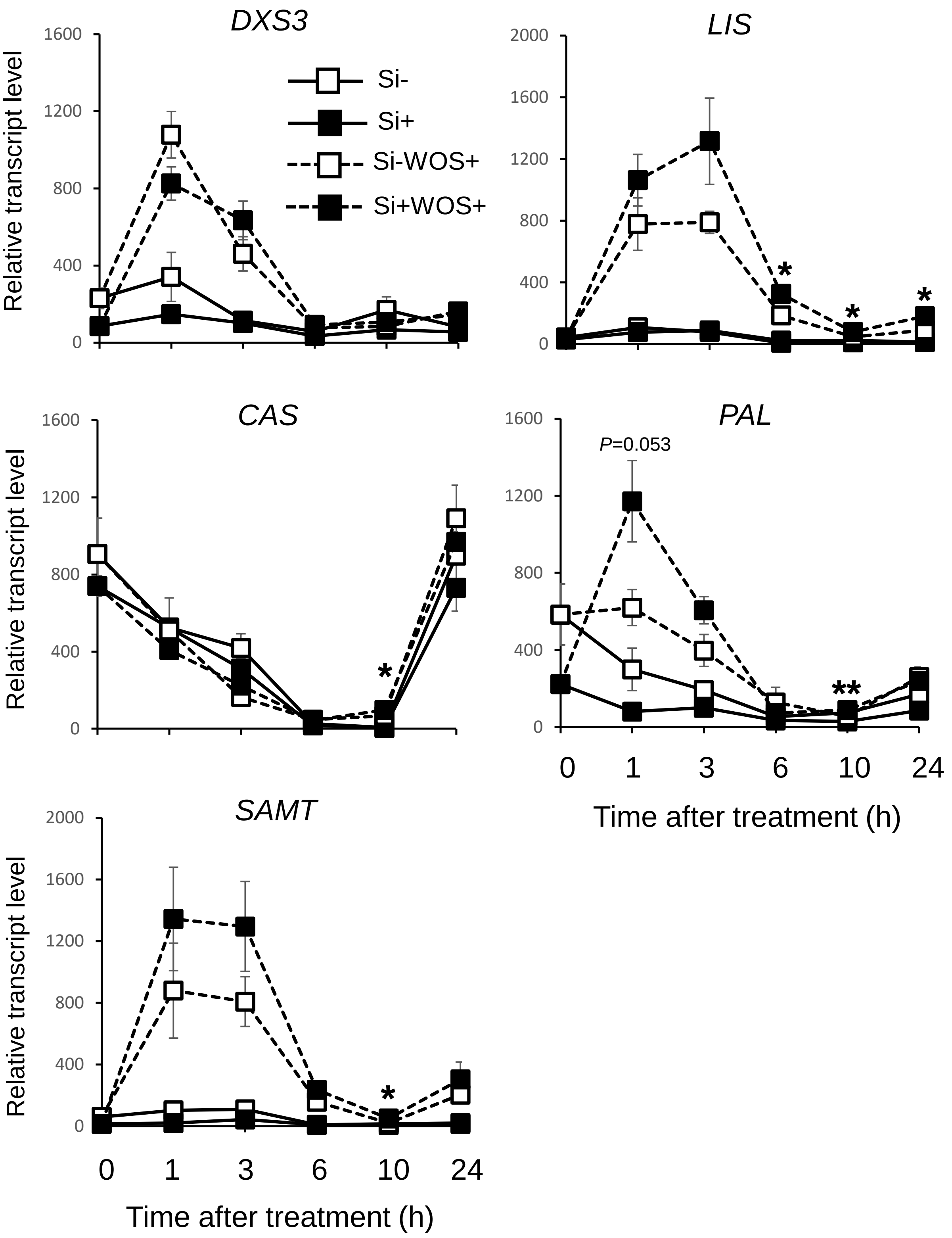


**Supplementary Figure 5. Relative transcript levels of VOC-related genes in rice plants after Si supplementation and WOS treatments.** WT rice plants were grown hydroponically with or without 0.5 mM Si (Control), subjected to wounds treated with oral secretions from *M. loreyi* (WOS). Gene expression was determined in control and WOS-treated fully expanded young and mature rice leaves by qRT-PCR. Data are means ± SE (n=4). Statistical differences within pairs of treatments (with and without Si) were analyzed by Student's t-test (*P<0.05; **P<0.01; no symbol, not significant).

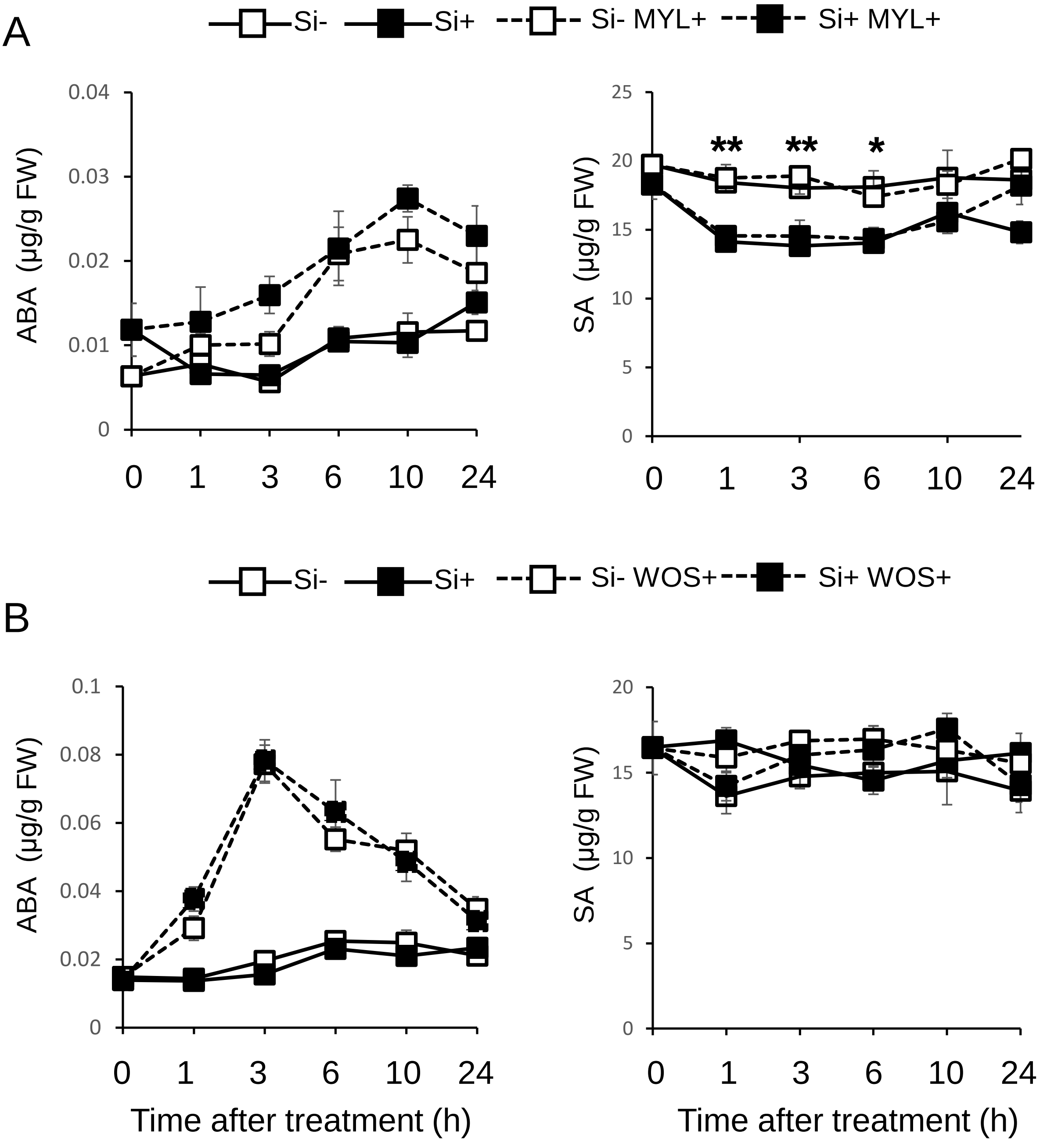


**Supplementary Figure 6. Si and herbivory-regulated accumulation of phytohormones in rice leaves.** Abscisic acid (ABA) and salicylic acid (SA) levels in mature rice leaves exposed to *Mythimna loreyi* feeding (MYL; A) or in fully expanded young and mature rice leaves elicited with wounds treated with oral secretions from *M. loreyi* (WOS; B). Data are means ± SE (n=4). Statistical differences within pairs of WOS treatments (with and without Si) were analyzed by Student's t-test (*P<0.05; **P<0.01; no symbol, not significant).
